# Supplementary material for: A genome-wide CRISPR-Cas9 knockout screen identifies FSP1 as the warfarin-resistant vitamin K reductase
Source: Nat Commun. 2023 Feb 14;14:828. doi: 10.1038/s41467-023-36446-8 (PMC9929328; doi:10.1038/s41467-023-36446-8)
Supplement: Supplementary file 1 — Supplementary Information [file 41467_2023_36446_MOESM1_ESM.pdf]

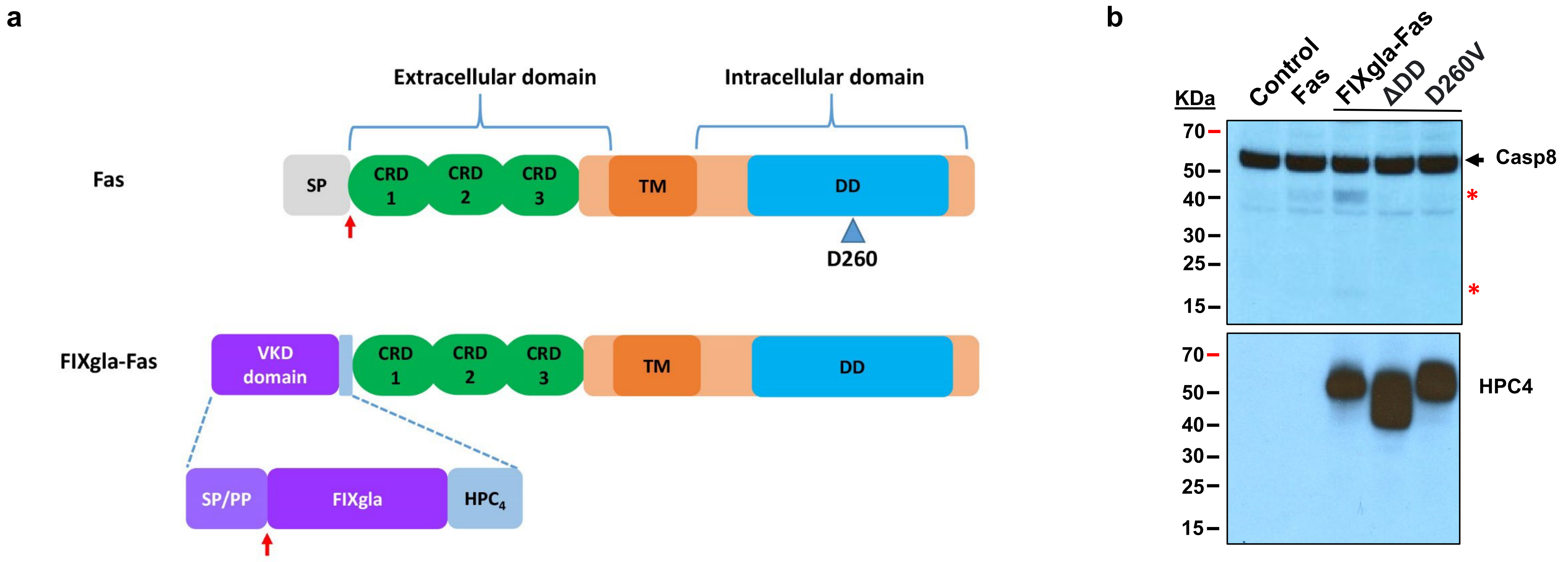

Supplementary Fig. 1 | Vitamin K-dependent apoptotic reporter protein. (**a**) Schematic diagram of the domain structure of the VKD apoptotic reporter protein FIXgla-Fas. The signal peptide (SP), propeptide (PP), and Gla domain of factor IX (FIXgla) is fused to the extracellular N-terminus of Fas with a HPC4 tag between the two fusion parts for detection purpose. Residue Asp260 within the death domain (DD), important for Fas-FADD interaction, is indicated by a triangle. CRD, cysteine-rich domain; TM, transmembrane domain. Arrowheads indicate positions of the cleavage of signal peptide or propeptide before mature protein is secreted. (**b**) Effect of FIXgla-Fas reporter protein and its mutants on caspase-dependent apoptosis detected by immunoblotting. The Fas, FIXgla-Fas, the D260V mutant, or FIXgla-Fas with its death domain (DD) deleted (ΔDD) constructs were transiently expressed in HEK293 cells. Transfected cells were incubated with 11 μM vitamin K for 48 hours before harvesting the cells for immunoblotting. Control: HEK293 cells only. Top: Activation of caspase-8 was probed using anti-caspase-8 antibody. Full-length caspase-8 was indicated by an arrowhead, activated caspase-8 was indicated by asterisks. Bottom: total reporter protein expression probed by anti-HPC4 tag antibody. Similar results were observed at least twice as shown in Fig. 1b.

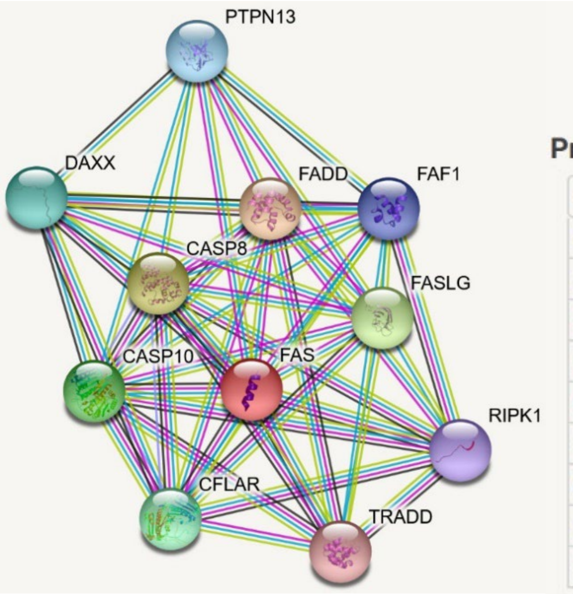

Predicted Functional Partners:

	Description	Neighborhood	Gene Fusion	Cooccurence	Coexpression	Experiments	Databases	Textmining	[Homology]	Score
FADD	FAS-associated death domain protein; Apoptotic adaptor molecule that rec...					●	●	●		0.999
CASP8	Caspase-8; Most upstream protease of the activation cascade of caspases.				●	●	●	●		0.999
FASLG	Tumor necrosis factor ligand superfamily member 6; Cytokine that binds to.					●	●	●		0.999
CASP10	Caspase-10; Involved in the activation cascade of caspases responsible foı.				●	●	●	●		0.997
CFLAR	CASP8 and FADD-like apoptosis regulator; Apoptosis regulator protein whic..				●	●	●	●		0.996
DAXX	Death domain-associated protein 6; Transcription corepressor known to rep..				●	●	●	●		0.995
PTPN13	Tyrosine-protein phosphatase non-receptor type 13; Tyrosine phosphatase ...					●	●	●		0.995
FAF1	FAS-associated factor 1; Potentiates but cannot initiate FAS-induced apopt					●	●	●		0.992
RIPK1	Receptor-interacting serine/threonine-protein kinase 1; Serine-threonine kin...				●	●	●	●		0.985
TRADD	Tumor necrosis factor receptor type 1-associated DEATH domain protein; 1..				●	●	●	●		0.983

Supplementary Fig. 2 | Network analysis of Fas receptor using the STRING protein-protein interaction network database with default settings. Of the ten proteins that interact with Fas in this network, the sgRNA targeting eight of the proteins (CASP8, CASP10, CFLAR, FADD, FAF1, PTPN13, RIPK1, and TRADD) were enriched in the genome-wide CRISPR-Cas9 loss-of-function VKD screening. Detailed sgRNA enrichment of these genes can be found in Supplementary Table 1.

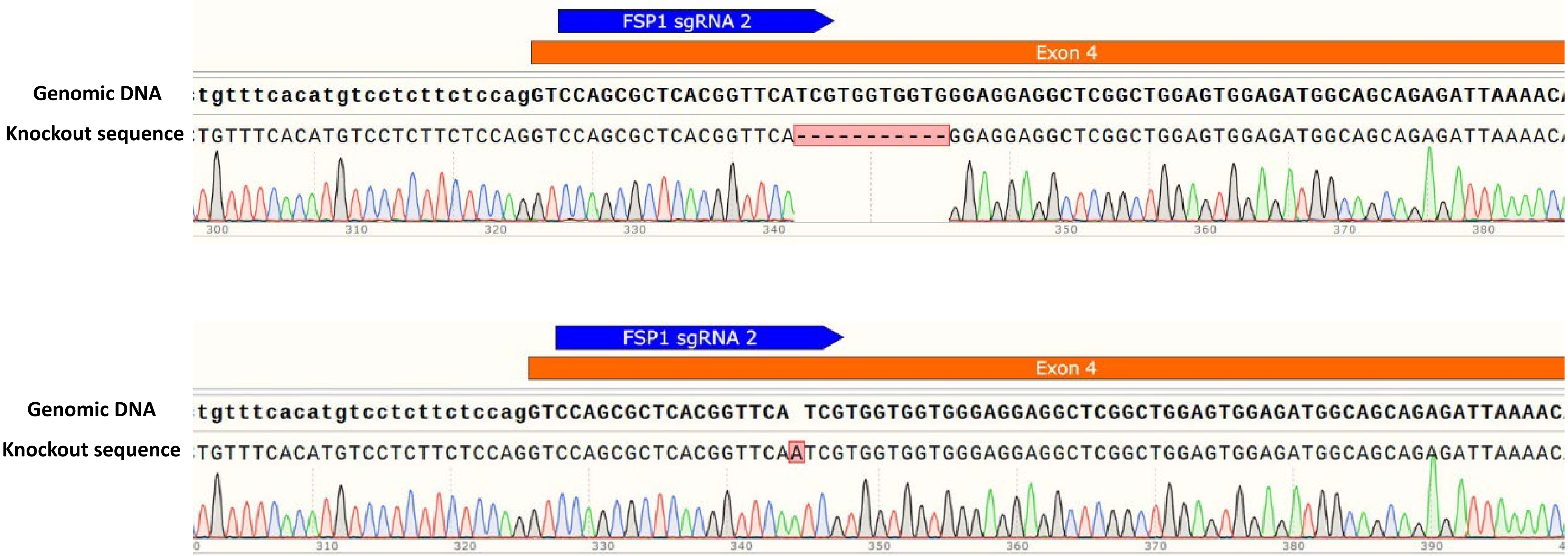

Supplementary Fig. 3 | Genomic DNA sequence analysis of FSP1 knockout HEK293 cells. The sgRNA of CCAGCGCTCACGGTTCATCG (FSP1-2) targeting exon 4 of *fsp1* was used to knock out the endogenous FSP1 in HEK293 cells. After single colonies were obtained, genomic DNA of the FSP1 knockout cells were extracted and used as a template for PCR amplification of the sgRNA targeted region. The sgRNA target sequence is indicated by blue arrows. Intron sequences are in lower case and exon sequences are in upper case. One of the single colonies has one allele has an 11-base deletion (top) while the other has a 1-base insertion (bottom) at the Cas9 cutting site.

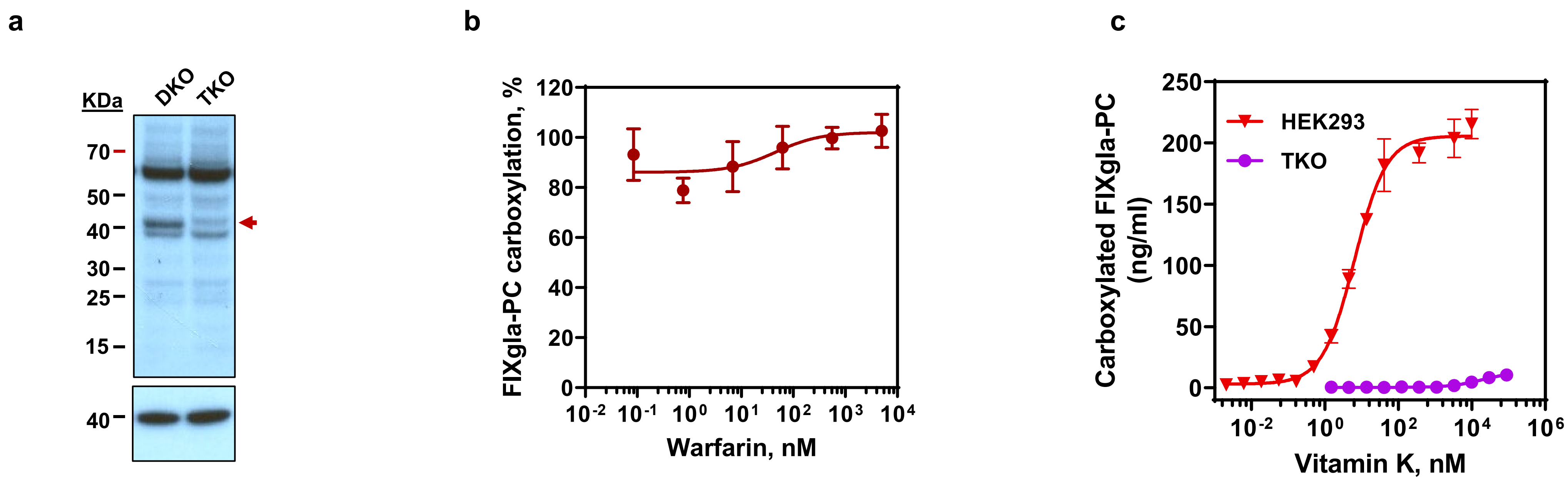

Supplementary Fig. 4 | Warfarin and vitamin K concentration titrations in HEK293 and TKO reporter cells. (**a**) Immunoblotting of DKO cells and these cells with their fsp1 gene knocked out (TKO). Full-length FSP1 is indicated by an arrowhead. Similar results were observed at least twice. Top: anti-FSP1, bottom: anti-GAPDH. (**b**) Inhibition of VKD carboxylation in TKO reporter cells. The TKO reporter cells were seeded in a 96-well plate and incubated with increasing concentrations of warfarin in the cell culture medium containing 11 μM vitamin K for the cell-based activity assay. The carboxylation activity of TKO cells without warfarin in the culture medium was normalized to 100%. Data are presented as mean ± SD of three independent experiments (n=3). (**c**) Vitamin K concentration titration of FIXgla-PC/HEK293 (HEK293) and TKO reporter cells. Reporter cells were seeded in 96-well plates and incubated with increasing concentrations of vitamin K for the cell-based activity assay. Carboxylated reporter protein FIXgla-PC in the cell culture medium were determined using ELISA without normalization. Data are presented as mean ± SD of three independent experiments (n=3).

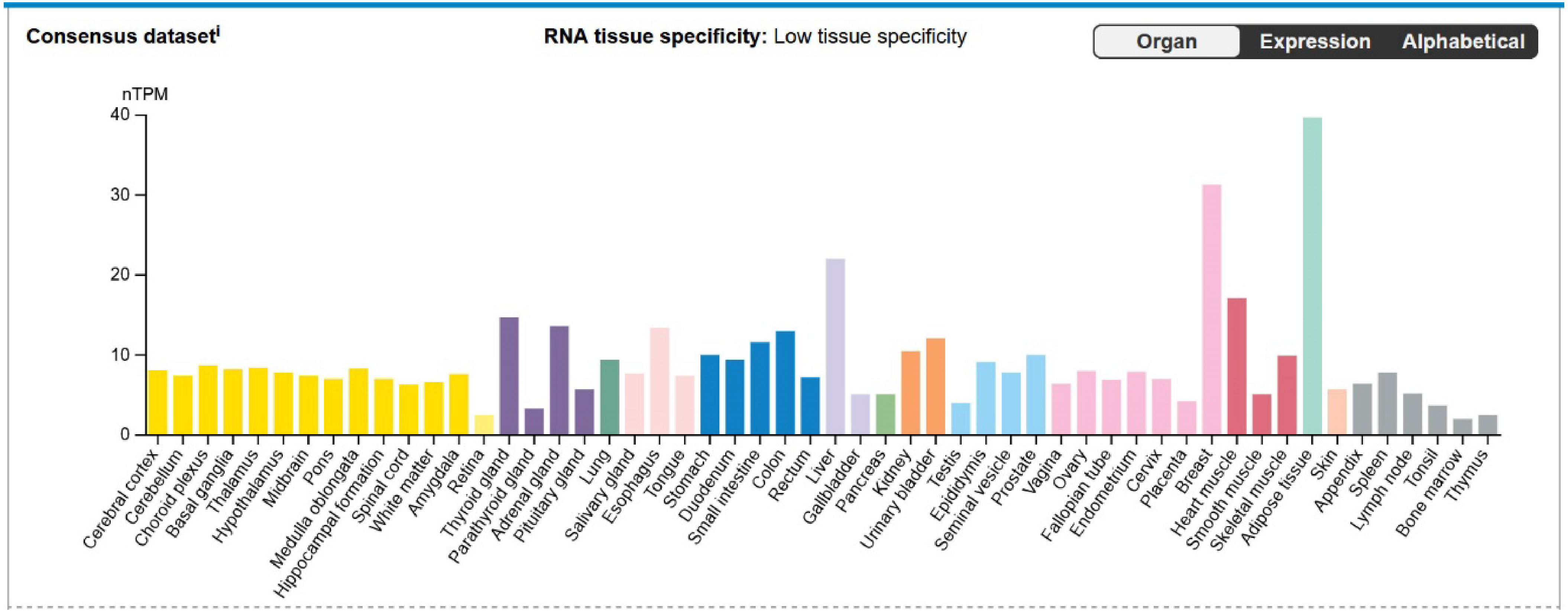

Supplementary Fig. 5 | Tissue specificity of FSP1 RNA from The Human Protein Atlas Database. A low tissue specificity for the FSP1 gene was determined by The Human Protein Atlas Database (https://www.proteinatlas.org/ENSG00000042286-AIFM2/tissue). AIFM2 was the protein name in the database while searching this protein.

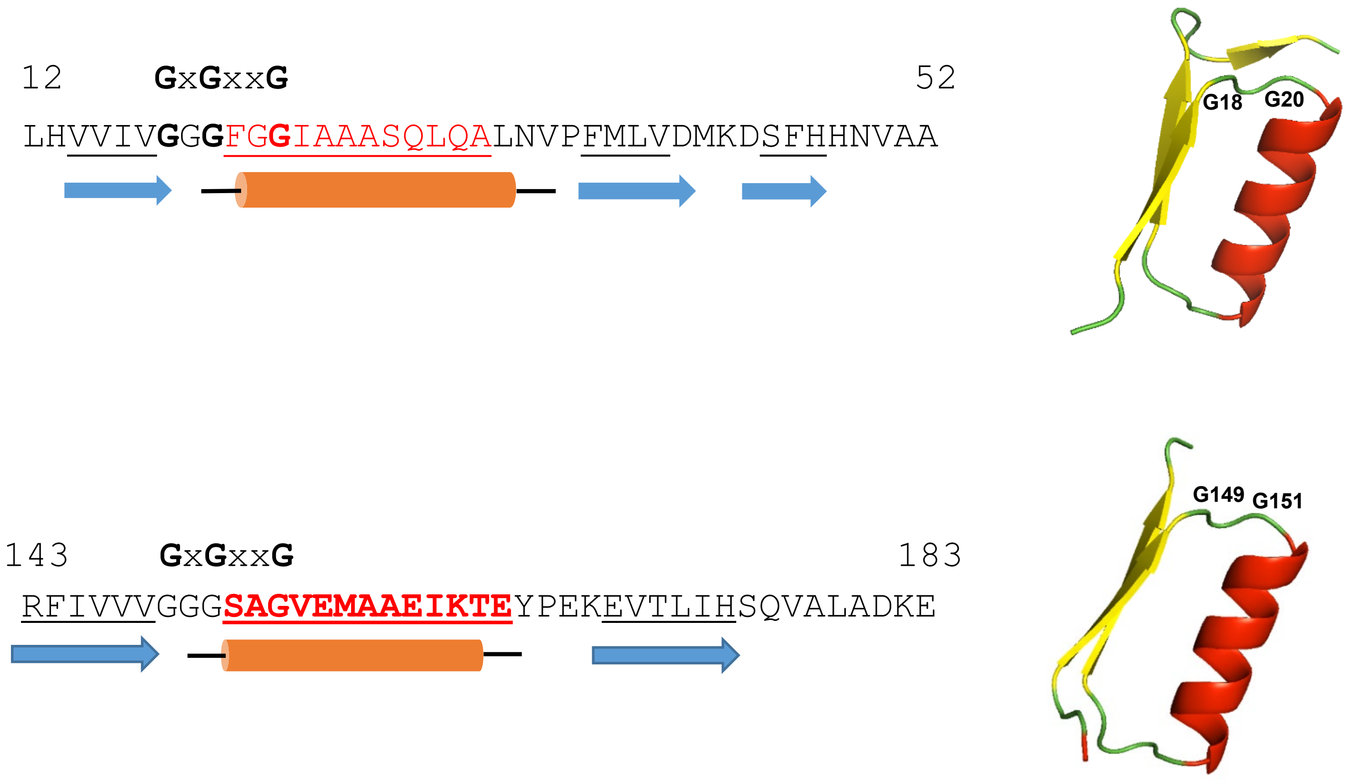

Supplementary Fig. 6 | Rossmann fold structural motifs identified in FSP1. Two Rossmann fold motifs were identified in FSP1 between residues 12 to 52 (top) and 143 to 183 (bottom). Rossmann fold motif structure typically contains an alpha helix flanked by two parallel beta strands. The loop connecting the first beta strand with the alpha helix contains the consensus sequence GxGxxG, which established direct interaction with the dinucleotide cofactor. Rossmann fold structures were obtain by Pymol using AlphaFold predicted model. Conserved glycine residues within the GxGxxG sequence that have been mutated in this study were indicated in the structure model.

```
   MG---S-------VV-VGGGFGG--AA--L--------L-D--D-FHHNV-ALRA-V--GFA--TFI-----F---F-Q-
            10        20        30        40        50        60        70        80
    *              *  **                  *
1  MGSQVSVESGALHVVIVGGGFGGIAAASQLQALNVPFMLVDMKDSFHHNVAALRASVETGFAKKTFISYSVTFKDNFRQG 80
2  MGSQISVDA-SVHVVVVGGGFGGIAAASQLQSWGVPFTLVDMRDSFHHNVAALRASVQSGFAKKTFISFAKTFGDSFRQD 79
3  MGSQVSVDVGSMHVVIVGGGFGGMAAASQLQAMNVPFTLVDMKDSFHHNVAALRASVESGFAKKTFISYSVTFKDNFRQG 80
4  MGSKVSVDA-SVRVVIVGGGFGGIEAALQLHSWEIPFLLVDMRDAFHHNVAALRASVDSGFAKKTFISFSVTFKDSFRQG 79
5  MGGQVSVDE-SVHVVIVGGGFGGIAAAQHLKHYGVPFMLIDVLDAFHHNVAALRASVQTGFARKTFIPYKETFGLNFLQG 79
6  MGSQVSVDMGAVRVVIVGGGFGGIAAASQLQTLNIPFMLVDMKDSFHHNVAALRASVESGFAKKTFISYSVTFKENFRQG 80
7  MGSKVSVDD-SVRVVIVGGGFGGMEAARLLQDWGIPFILVDMRDSFHHNVAALRASVQSGFAKKTFISFSETFKDSFQQG 79
8  MGGQISVDK-SVQVVIVGGGFGGIEAARKLKCWGVPFVLVDMRDAFHHNVAALRAAVQSGFAKKTFISFSKTFGGSFRQG 79
9  MGSKLSIDE-NVHVVVVGGGFGGIAAAIQLKSLGVPYTLIDMKDAFHHNVGALRASVQSGFAKTTFISYSATFNGNFKQG 79
10 MGGKLSVDE-NVHVVVVGGGFGGIAAAKQLKCHGVPYTLIDIRDAFHHNPGALRASVESGFAKKTFISFRETFKENFKQG 79
11 MGSKVSVEE-SVRVVIVGGGFAGIAAATQLKSFGIPFVLVDLKDAFHHNVAALRASVESGFARKTFISYKDTFQDNFIQG 79
12 MGSQVSVDTGAVHVVIVGGGFGGIAAASQLQALNVPFMLVDMKDSFHHNVAALRASVESGFAKKTFISYSATFKDNFRQG 80
13 MGGQVSIDE-TVHVVIVGGGFGGIAAAQHLKHHGVPFMLIDILDAFHHNVAALRASVQAGFAKQTFIPYKETFGLNFLQG 79
14 MGSRLSLDG-SVRVVVVGGGFGGTAAASLLKSWAVPFVLVDVRDAFHHNVAALRAAVESGFAKKTFISYSVTFGDSFRQG 79
15 MGSRLSVDD-SVRVVIVGGGFGGIAAASQLKSWAVPFVLVDMRDAFHHNVAALRASVESGFARKTFISYSVTFGDSFRQG 79
16 MGSKLSVDD-SVCVVIVGGGFGGIAAARQLKSWGIPFVLVDMRDAFHHNVGALRASVESGFAKKTFISYSVTFEGSFRQA 79
17 MGGQVSTAD--VHVVVVGGGFGGIAAALRLQSRGLAFTLIDLRDSFHHNVAALRASLQPGFAKRTFIPYANTFGDSFVQG 78
18 MGSQVSVDMGAVHVVVVGGGFGGIAAASQLQALNIPFTLVDMKDSFHHNVAALRASVESGFAKKTFISYSATFKENFRQG 80
19 MGSRLSVDD-SVRVVVVGGGFGGAAAASQLKSWGIPFVLVDVRDAFHHNVGALRAAVQSGFAKKTFISYSVTFGDSFRQG 79
20 MGSQVSVDVGAVHVVIVGGGFGGIAAASQLQALNIPFMLVDMKDSFHHNVAALRASVESGFAKKTFISYSVTFKENFRQG 80
21 MGSQVSMDAGAVHVVIVGGGFGGIAAASQLQALNIPFVLVDMKDSFHHNVAALRASVESGFAKKTFISYSVTFKENFRQG 80
22 MGSQVSMDKGAVHVVIVGGGFGGIAAASQLQALNIPFLLVDMKDSFHHNVAALRASVESGFAKKTFISYSVTFKENFRQG 80
23 MGSQVSADVGPVHVVIVGGGFGGMAAASQLQALNIPFTLVDMKDAFHHNVAALRASVESGFAKKTFISYSVTFKDNFRQG 80
24 MGSKLSVDD-SVRVVIIGGGFGGIEAARQLKSWGIPFVLVDMRDAFHHNVGALRASVESGFAKKTFISYSVTFEESFRQA 79
25 MGSKVSVDD-SVRVVIVGGGFGGIEAACQLQCWGVPFVLIDMRDAFHHNVAALRASVQSGFAKKTFISFSVTFKDSFRQG 79
26 MGSQVSADTGALHVVIVGGGFGGIAAASQLQALNIPFLLVDMKDSFHHNVAALRASVESGFAKKTFISYSVTFKDNFRQG 80
27 MGSQVSMDKGAVHVVIVGGGFGGIAAASQLQALNIPFLLVDMKDSFHHNVAALRASVESGFAKKTFISYSASFKENFRQG 80
28 MGSKLSVDD-SVRVVIVGGGFGGIAAASQLKSWAIPFVLVDMRDAFHHNVGALRASVESGFAKKTFISFSVTFGDSFRQG 79
29 MGSQVSMDTRGVHVVIVGGGFGGIAAASQLQALNVSFVLVDMKDFFHHNVAALRASVESGFAKKTFISYSVSFKESFRQG 80
30 MGGSLSIDD-GVHVVIVGGGFGGIAAAQQLKYYRVPFTIIDLRDAFHHNVASLRASVQSGFAKQTFIPFRETFGDSFLQG 79
```

```
   -V---D-----V-L--------SHLIL-TG--G-FPGK---------AI--YE--------------VGGG--GVEMAAE
            90        100       110       120       130       140       150       160
                                                                     *  *
1  LVVGIDLKNQTVLLQGGEALPFSHLILATGSTGPFPGKFNEVSSQQAAIQAYEDMVRQVQRSRFIVVVGGGSAGVEMAAE 160
2  LVVGLDLEKQNVLLQGGEALSYSHLILATGSTGPFPGKFNQISNLQSAIEAYEVMVKQVQNSQHIVVVGGGSAGVEMAAE 159
3  VVVGIDLKNQTVQLQGGEALPFSHLILATGSTGPFPGKFNQVSSQQAAIQAYEDIVRQVQSSQVIVVVGGGSAGVEMAAE 160
4  TVVGIDLDKQHVLLNDGEEIFFSHLILATGSDGPFPGKFNQVIDMQAAIQTYEDMAKEVQKAPRIVIVGGGSAGVEMAAE 159
5  RVIRIDTETQTVVLDNGKEVRYSHLILCTGTTGSFPSKHNSVDTYKSAIQKYEDFFHVIKEANAIVVVGGGTTGVEMAAE 159
6  LVVEIDVQNQMVLLEDGEALPFSHLILATGSTGLFPGKFNQVSSWELAIQAYEDMVKQVQRSQSVVVVGGGSAGVEMAAE 160
7  KVVEIDLEKHQVLLNDGKVLSFSHLILATGSEGPFPGKFNELINMEMAIQAYENIVKEVEKAERVVIVGGGSAGVEMAAE 159
8  LVVGIDLEKRHVLLNDGEHLCYSHLILATGSDGPFPGKFDKPMDMQSAIQTYEDMVQEVEKSPRIVIVGGGSAGVEMAAE 159
9  KVMDIDTQKQCVILEDREEVCFSHLILATGSDGPFPGKFNKSVTKESAIQMYENLVQEVQKAERIVVVGGGSAGVEMAAE 159
10 KVVDIDLEKQNVVLDSGEVVHFSHLILATGSTGPFPGKFNEPVSMESAIQMYEDLVKKIQEASRIVLVGGGSAGVEMAGE 159
11 KVVGINLQTQRVILESNEELQFSHLIIATGSNGPFPGKINNVISKDQAIQVYEDLVKEIQKAKHVVVVGGGSAGVEMAAE 159
12 KVIGIDLKNRMVLLQGGEALPFSHLILATGSTGPFPGKFNEVSCQQAAIQAYEDMVKQIQRSQFIVVVGGGSAGVEMAAE 160
13 RVIQIDSETQTVVLDNGKQVRYSHLILCTGTSGHFPGKHNSVATYQSAIQKYEDFVNVIQEANTVVVVGGGTTGVEMAAE 159
14 KVVAIDPGRQQVVLSDGEELHYSHLILATGSDGPFPGKFNQVIDMESAIQTYEDMVKEIEKSQRILVVGGGAAGVEMAAE 159
15 KVVGIDPERQQVLLSDGEELHYSHLILATGSDGPFPGKFNKVIDMESAIQTYEDMVKEIEKSERILVVGGGAAGVEMAAE 159
16 KVVGIDPLRQKVLLNDDEELFYSHLILATGSDGPFPGKLNQIIDMEAAIRTYEDLVQELQKSPRIVIVGGGAAGVEMAAE 159
17 RVERIDTGRQAVVLEGGREIQFTHLILCTGTDGTFPGRFNTVASHQSAVQSYEDFVGQVQAADSVLVIGGGSTGVEMAAE 158
18 LVVEIDLQNQTVLLEDGEALPFSHLILATGSTGLFPGKFNQVASLEVAIQAYEDMVKQVQRSQSVVVVGGGSAGVEMAAE 160
19 QVVGIDPERQQVLLSDGEELHYSHLILATGSDGPFPGKFNKVIDMESAIRSYEDMNKEVEKSQRILVVGGGSAGVEMAAE 159
20 LVIEIDLQNQMVLLEDGEALPFSHLILATGSTGLFPGKFNQVSSREAAIQAYEDMVKQVQRSQSVVVVGGGSAGVEMAAE 160
21 LVVEIDLKNQTVLLEDGQALPFSHLILATGSTGLFPGKFNQVSSQQMAIQAYEDMVTQVQRSQSIVVVGGGSAGVEMAAE 160
22 LVVGIDLKNQTVLLEDGEALRFSHLILATGSTGLFPGKFNQVSSQQVAIQAYEDMVTQVQRSQSIVVVGGGSAGVEMAAE 160
23 VVVGIDVKNQTVQLQGGEALPFSHLILATGSTGPFPGKFNQVSSQQAAIQAYEDLVRQVQSSQFIVVVGGGSAGVEMAAE 160
24 KVVGIDLLRQKVLLNDDEELFYSHLILATGSDGPFPGKFNQIVDMETAIQTYEDMVKELQKSPRIVIVGGGAAGVEMAAE 159
25 LVTGIDLEKQCVLLNDGEEVFFSHLILATGSDGPFPGKFEKPIDMEPAIQTYEDMVKEVEKAPRIVVVGGGSAGVEMAAE 159
26 QVVGIDLKNQMVLLQGGEALPFSHLILATGSSGPFPGKFNKVSCQQAAIQAYEDMVKQVQRSQFIVVVGGGSAGVEMAAE 160
27 LVVGIDLKNQTVLLEDGEALRFSHLILATGSTGLFPGKFNQVSSQQVAIQAYEDMVTQVQCSQSIVVVGGGSAGVEMAAE 160
28 KVVGIDPGKQQVLLSDGEELHYSHLILATGSDGPFPGKFNKVIDMESAIQTYEDMVKEIEKSQRILVVGGGSAGVEMAAE 159
29 LVVEIDLKNQTVLLEDGEVLPFSHLILATGSTGLFPGKLNQVCSQQEAIQAYEDIVKQVQHSQSIVVVGGGSAGVEMAAE 160
30 RVARVDTSAQIVVLEDGKEVHYSHLILCTGTDGPFPGKYNTLASYQTAIEKYEEFVKEVQAAGTVLVVGGGSTGVEMAAE 159
```

```
   --T----K-V-L-H-----AD--LL--V----K--L----V-L-L---V-N------N---------T-K---------V
        170       180       190       200       210       220       230       240
1  IKTEYPEKEVTLIHSQVALADKELLPSVRQEVREILLRKGVQLLLSERVSNLEELPLNEYREYIKVQTDKGTEVAT-NLV 239
2  LKTDYPEKEVTLIHSKVPLADPELLPCVRQEVKEILLQKGVELLLSERVSNLEELPLNECRDSIQVKTDKGTQLAA-NLV 238
3  IKTEYPEKEVTLIHSQVALADKELLPCVRQEVKEILLRKGVQLLLSERVSNLEELPVNEYREYIKVQTDKGTEVAT-NLV 239
4  VKTMYPTKEVALIHSKIALADEELLPRVRQEVKETLIHEGVNLFLGQRVDNLHELTLHQFKENMVVKTDKGTEMVT-DMV 238
5  IKTEFHDKKVVLIHPREEVADPELLPCVKEQAKQVLLEKGVELLLGQKVSNLEELELNVCRSGMVVKTNKNEQVTT-DLV 238
6  VKTEFPEKEVTLIHSQVALADKELLPCVRQEAKEILLQKGVQLLLSERVSNLEDLPLNEYRERIQVHTDKGTEVAT-NLV 239
7  VKTTYPNKEVTLVHSKMALADGGLLPRVREAVKETLTQQGVHLLLGQKVSNLHMLSLNQFKENMMVKTDKGTEVAA-DLV 238
8  VKTKYPNKEVTLIHSKVALADAEVLPRVRQEVKESLLKEGVHLLLSQKVENLQSLTLNQFKENMIVKTDKGTEIVT-DMV 238
9  IKSDYPQKEVTLIHSKVALADVELLPSVRQTVKEILLRKGVKLLLSQRASNLTQLKLNEVQQEMSVETDKGTTVTA-DLV 238
10 IKTDYPDKEVILIHSKIALADIELLPSVRQGVKEILVKKGVQLELGQKVTNLKELVTNETKNDQKVLTDKGLEFNA-DMV 238
11 VKTDYPEKEVTLVHSKVALADVQLQPKVRRTVKEILLSKGVRLILAQKVTNLDQVTSNVAQENTVLQLDKNSEVVTCDLV 239
12 IKTEYPEKEVTLIHSRVPLADKELLPCVRQEVKEILLRKGVQLLLSERVSNLEELPRNEYREYIKVETDKGTEVAT-NMV 239
13 IRTEFTDKKVILIHPREEVADPDLLPSVKEQAKQVLLEKGVELLLGRKVTNLDELELNVCRKGMVIKTNKNEQVTA-DLV 238
14 IKTEYPGKEIILIHSKTALADVELLPSVRQVVKEILLRKGVRLLLSEKVSDIENLRPNQFQKDMVVRTEKGTEVVV-DMV 238
15 IKTEYPAKEVTLIHSKIALADVELLQSVRQEVKEILLRKGVRLLLSEKVSNVENLTTNQFQKDMVVRTEKGTEVVV-DMV 238
16 IKTEYPAKEVTLIHSKTALADVELLPSVRQEVKEILLRKQVHLLLSERVQNMHTLTLNQFQENMAVKTDKGREVAA-DMV 238
17 IRTEYPDKKVVLVHSRMQLADPDLLPIVRYQAKEVLLEKGVEVLLGHKVSNLSELKLNATTKNMEVVTDKGERIKT-DLI 237
18 IKTEFPEKEVTLIHSQVALADKELLPCVRQEAKEILLQKGVQLLLSERVNNLEQLPLNEYRERIQVHTDKGTEVAT-NLV 239
19 IKTEYPAKEVTLIHSKMALADVELLPSVRQEVKEILLRKGVHLLLGERVSNLEKFTPNCFQKDMVVRTEKGTEITV-DMV 238
20 VKTEFPEKEVTLIHSQVALADKELLPCVRQEAKEILLQKGVQLLLSERVSNLEDLPLNEYRECIQVHTDKGTEVAT-NLV 239
21 IKTEYPEKEVTLIHSKMALADTELLPCVRQEVKEILLRKGVQLLLSERVSNLEALPVNEHRECIKVQTDKGTEVDA-NLV 239
22 IKTEYPEKEVTLIHSQMALADKELLPCVRQEAKEILLRKGVQLLLSERVSNLEELPLNEYQECIKVQTDKGTEVAA-NLV 239
23 IKTEYPEKEVTLIHSKVPLADQELLPCVRQEVKEILLRKGVQLLLSERVSNLEDLPLNEYREYIKVQTDKGTEVAT-NLV 239
24 IKTEYPAKEVTLIHSKIALADVELLPSVRQMVKEILLKKEVRLLLSDRVSNLHMLTLNRFQENMAVKTEKGTEVAA-DMV 238
25 ISTKXPSKEVTIIHSKPALADAELLPRVRQEVKETLIQEGVHLLLNHKVSNLDKLTLNQFKENIVVETDKGTEVVT-DMV 238
26 IKTEYPDKEVTLIHSQVPLADKELLPCVRQEVKEILLRKGVQLLLSERVSNLEEIPLNEYREYIKVQTDKGTEVAT-NLV 239
27 IKTEYPEKEVTLIHSQMALADKELLPCVRQEAKEILLRKGVQLLLSERVSNLEELPLNEYRECIKVQTDKGTEVAA-NLV 239
28 IKTEYPAKEVTLIHSKIALAEVELLHSVRQEVKEILLRKGVRLLLSERVSNLENFTPNQFQKNMVVMTEKGTEVIA-DMV 238
29 IKTEHPEKEVTLIHSQVALADKELLPCVRQEAKEILLRKGVQLLLSERVSNLEELPVNEYRECIKVQTDKGTEVAT-NLV 239
30 IKTEFPDKKVILIHSRNVLADPELLPSVREQAKEVLLQKGVELLLEQKVTNLNDLELNVTRKDIVIKTDKDTEITA-DLV 238
```

```
   --C-G---NS-AY---F---LASNGAL-VNE-LQVEGY-NIYAIGDCADVKEPKMAYHAGLHANVAVTNI-NSL-QKPLK
            250       260       270       280       290       300       310       320
1  ILCTGIKINSSAYRKAFESRLASSGALRVNEHLQVEGHSNIYAIGDCADVRTPKMAYLAGLHANIAVANIVNSVKQRPLQ 319
2  ILCNGIKINSSAYSSALGDQMADNGALMVNDHLQVQGFSNIYAIGDCANVKEPKMAYHAGLHANVVVTNIVNSLKQKPLK 318
3  IPCNGIKVNSSAYCSAFESRLASNGALQVNEYLQVEGYSNVYAIGDCADVKEPKMAYHAGLHANIVTTNIINSMKQRPLK 319
4  ILCTGIKVNSSAYRSSFINKLASNGALKVNDHLQVEGYDNIYAIGDCADVKEPKMAYHAGLHADVAVTNIINSLTQKPLK 318
5  ICCTGSKINSEAYRSSMSSCLAENGALKVNKHLQVEGFDNVYAVGDCANLSEPKMAYHAGLHAGVAATNIINSLSGKALT 318
6  IVCNGIKVNSSAYHSAFDGHLASNGALRVNEYLQVEGYSHIYAIGDCADVKEPKMAYHAGLHASVAVANIVNSRKQRPLK 319
7  ILCTGIKINSSAYSSAFRDQLANNGALKVNDYLQVVGYDNIYAIGDCSDVKEPKMAYHAQLHANVVVTNIINSLTEKPLK 318
8  ILCTGIKVNSAAYSGAFSDKLASNGALQVNEYLQVKGYDNIYAIGDCADVNEPKMAYHAGLHADVAVTNIVNSLTQKPLK 318
9  ICCTGIKVNSSAYSNALKLKLADNGALRVNEHLQVEGSENIYAIGDCADVKEPKMAYHAELHAKVAVKNIVNSLTQKPLA 318
10 ICCTGIKINSSAYENSFKDAIVKNGALMVNKHLQVEGMENIYAIGDCADVKEPKMAYHAGLHGAVAVDNIINTLKEKPLK 318
11 LCCTGYKISSSSYSSAFGDKLAEDGALIVNDYLQVQGHANVYAVGDCAYINEPKMAYYAGIHARVAATNVRNSLIGKSLK 319
12 IVCNGIKINSSAYRSAFESRLASNGALKVNEFLQVEGYSNIYAIGDCADTKEPKMAYHAGLHANVAVANIVNSMKQRPLK 319
13 ICCTGNKINSEAYRSSLTTCMAESGALKVNQHLQVEGFDNVYAVGDCANLDEPKLAYHAGLHAAVAATNIISSLTGKCLT 318
14 VLCTGIKINSSAYAAAFGDKMASDGALKVNKHLQLEGYENIYAIGDCADLKEPKMAYHAGLHANVVVTNIINSLTQKPLK 318
15 VLCTGIKINSSAYATAFGDKLASNGALNVNKHLQLEGYDNIYAIGDCANLKEPKMAYHAELHANIVVSNIINSLTHKPLK 318
16 ILCTGIKINSSAYSSAFGEKLASNGALKVNQYLQVEGYDNIYAIGDCADVKEPKMAYHAGLHANIVVTNIINSLTQKPLK 318
17 FCCTGLRVNSSAYKSSFSDHMTNSGALKVNEHLQVEGFSNVFAIGDCNNVNEAKTAYNAELHAGIAVGNIANSVNGKRLT 317
18 IVCNGIRINSSAYRSAFESHLAGNGALRVNEYLQVEGCSHVYAIGDCADVKEPKMAYHAGLHASVAVANIVNARKQRPLK 319
19 ILCTGIKINSSAYGSAFGDKMASNGALRVNQHLQLEGYENIYAIGDCADLKEPKMAYHAGLHANVVVTNIVNSLKNKSLQ 318
20 IVCNGIKINSSAYHSAFDSHLASNGALRVNEHLQVEGCSRVYAIGDCADVKEPKMAYHAGLHAGIAVANIVNSMKQRPLK 319
21 IVCNGIKINSAAYRSAFGDRLASNGALRVNEYLQVEGYSHIYAIGDCADVREPKMAYHASLHANVAVANIVNSMKQRPLK 319
22 IVCNGIKINSSAYRSAFDDGLASNGALKVNEYLQVEGCSHVYAIGDCADVKEPKMAYHAGLHANVAVANIINSMKQRPLK 319
23 IVCNGIKINSSAYCSAFESRLASNSALRVNEYLQVEGYSNIYAIGDCADVKEPKMAYHAGLHANIVTTNIVNSMKQRPLK 319
24 ILCTGIKINSSAYSSAFGDKLASNGALKVNQYLQVEGYDNIYAIGDCADVKEPKMAYHAGLHANIVVTNIINSLTQKPLK 318
25 ILCTGIKVNSSAYRSAFSDKLAKNGALQVNEHLQVKGYKNIYAIGDCAXVREPKMAYHASLHADVAVANIVNSLTNKPLK 318
26 ILCNGIKINSSAYSSAFESRLASNGALRVNEFLQVEGYSNIYAIGDCADVKEPKMAYHAGLHANVAVTNLVNSMKQRPLK 319
27 IVCNGIKINSSAYRSAFDDGLASNGALKVNEYLQVEGCSHVYAIGDCADVKEPKMAYHAGLHANVAVTNIINSMKQRPLK 319
28 ILCTGIKINSSAYASAFADKLASNGALKVNQHLQLEGYENIYAIGDCADLKEPKMAYHAGLHANIVVTNIINSLTHKSLK 318
29 IVCNGIKINSFAYRRAFASQLASNGALPVNEYLQVEGCSHIYAIGDCADVKEPKMAYHAGLHANVAVANIINSTQQRPLK 319
30 ICCTGNKINSTAYSSSLSECLAEDGSLNVNEHLQVTGFQNVYAVGDCANIKEPKMAYHAGLHGGVAATNIINNLSGKPLT 318
```

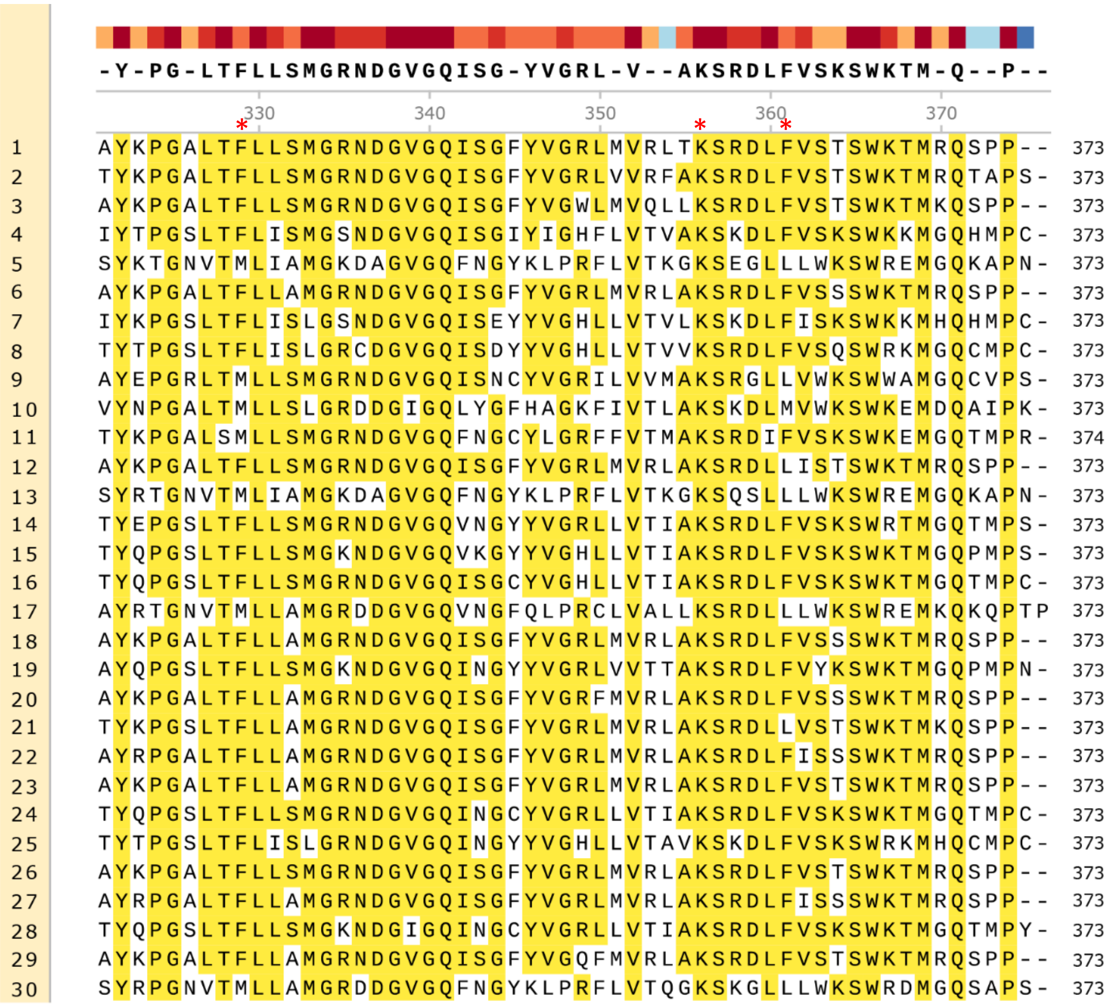

1. tr H2Q210 H2Q210_PANTR Apoptos is induc ing factor mitochondria assoc iated 2 O S=Pan troglodytes OX=9598 GN=AIFM2 PE=2 SV=1
2. tr A 0A4X2LCH5 A 0A4X2LCH5_VOMUR Apoptos is induc ing factor mitochondria assoc iated 2 O S=Vombatus urs inus OX=29139 GN=AIFM2 PE=4SV=1
3. tr G3TFJ2 G3TFJ2_LOXAF Apoptos is induc ing factor mitochondria assoc iated 2 O S=Loxodonta africana OX=9785 GN=AIFM2 PE=4 SV=1
4. tr G1KUD7 G1KUD7_A NOC A L-amino-acid oxidase O S=A nolis carolinensis O X=28377 GN=aifm2 PE=3 SV=2
5. tr E7FFC4 E7FFC4_DANRE A poptos is-induc ing factor mitochondria-assoc iated 2 OS=Danio rerio O X=7955 GN=aifm2 PE=1 SV=1
6. tr E2RQW8 E2RQW8_CANLF Apoptos is induc ing factor mitochondria assoc iated 2 O S=Canis lupus familiaris O X=9615 GN=AIFM2 PE=4 SV=1
7. tr A 0A6J1UCF5 A 0A6J1UCF5_9SAUR L-amino-acid oxidase OS=Notechis scutatus OX=8663 GN=A IFM2 PE=3 SV=1
8. tr A 0A670I7A9 A 0A670I7A9_PODMU L-amino-acid oxidase OS=Podarcis muralis OX=64176 GN=A IFM2 PE=3 SV=1
9. tr M3XJX5 M3XJX5_LATCH P yr_redox_2 domain-containing protein O S=Latimeria chalumnae O X=7897 GN=A IFM2 P E=4 SV=1
10. tr A 0A4W3HBP0 A 0A4W3HBP0_CALMI Pyr_redox_2 domain-containing protein OS=Callorhinchus milii OX=7868 GN=aifm2 PE=4 SV=1
11. sp B4F6I3 FSP1_XENTR Ferroptosis suppres sor protein 1 OS=Xenopus tropicalis O X=8364 GN=aifm2 P E=2 SV=1
12. sp Q 8BUE4 FSP1_MOUSE Ferroptos is suppressor protein 1 OS=Mus musculus OX=10090 GN=Aifm2 PE=1 SV=1
13. tr A 0A672QUN3 A0A672QUN3_SINGR Apoptos is induc ing factor mitochondria assoc iated 2 O S=Sinocyc locheilus grahami O X=75366 GN=aifm2P E=4 SV =1
14. s p B5FXE5 FSP 1_TA EGU Ferroptos is suppres sor protein 1 O S=Taeniopygia guttata O X=59729 GN=A IFM2 P E=2 SV=1
15. tr E1BR24 E1BR24_CHIC K P yr_redox_2 domain-containing protein O S=Gallus gallus O X=9031 GN=A IFM2 P E=4 SV=1
16. tr K7G371 K7G371_PELSI Pyr_redox_2 domain-containing protein OS=Pelodiscus s inensis OX=13735 GN=AIFM2 PE=4 SV=1
17. tr A0A3B5KF37 A0A3B5KF37_TAKRU Pyr_redox_2 domain-containing protein O S=Takifugu rubripes O X=31033 GN=aifm2 PE=4 SV=1
18. tr A0A6G1AF34 A0A6G1AF34_CROCR A IFM2 factor (Fragment) O S=Crocuta crocuta OX=9678 GN=A ifm2 PE=4 SV=1
19. tr A0A7K4LBN8 A 0A7K4LBN8_9AVES A IFM2 factor (Fragment) O S=Crypturellus undulatus O X=48396 GN=Aifm2 PE=4 SV=1
20. tr A 0A2U3ZUK2 A0A2U3ZUK2_ODORO apoptos is-induc ing factor 2 OS=Odobenus rosmarus divergens O X=9708 GN=AIFM2 PE=4 SV=1
21. tr A0A6P5AS70 A0A6P5AS70_BOSIN apoptosis-inducing factor 2 O S=Bos indicus O X=9915 GN=AIFM2 PE=4 SV=1
22. tr A0A6I9IIM4 A 0A6I9IIM4_VICPA apoptos is-induc ing factor 2 isoform X1 OS=Vicugna pacos OX=30538 GN=AIFM2 PE=4 SV=1
23. tr A0A6I9J4K1 A 0A6I9J4K1_CHRAS apoptosis-inducing factor 2 O S=Chrysochloris asiatica OX=185453 GN=AIFM2 PE=4 SV=1
24. tr A 0A8C3ISU4 A0A8C3ISU4_CHRPI Apoptos is induc ing factor mitochondria assoc iated 2 O S=Chrysemys picta bellii O X=8478 GN=AIFM2 PE=4SV=1
25. tr A 0A8D0DNU7 A0A8D0DNU7_9SAUR Apoptos is induc ing factor mitochondria assoc iated 2 O S=Salvator merianae OX=96440 GN=AIFM2 PE=4SV=1
26. tr A 0A8C5Z5X0 A 0A8C5Z5X0_MARMA Apoptos is induc ing factor mitochondria assoc iated 2 O S=Marmota marmota marmota O X=9994 GN=AIFM2 P E=4 SV =1
27. tr A 0A8B7K828 A0A8B7K828_CAMFR apoptosis-inducing factor 2 isoform X1 OS=Camelus ferus OX=419612 GN=A IFM2 PE=4 SV=1
28. tr A 0A8B7IJL1 A 0A8B7IJL1_9AVES apoptosis-inducing factor 2 isoform X1 OS=Apteryx mantelli mantelli OX=202946 GN=AIFM2 PE=4 SV=1
29. tr A 0A671EWY5 A0A671EWY5_RHIFE Apoptos is induc ing factor mitochondria assoc iated 2 O S=Rhinolophus ferrumequinum O X=59479 GN=AIFM2 P E=4 SV =1
30. tr A 0A6J2V437 A 0A6J2V437_CHACN apoptosis-inducing factor 2 isoform X2 OS=Chanos chanos OX=29144 GN=aifm2 PE=4 SV=1

Supplementary Fig. 7 | FSP1 sequence alignment from 30 diverse species using SnapGene. Conserved sequences are highlighted in yellow. Consensus threshold was set as 95%. Residues mutated for functional study in this paper are indicated by asterisks. The GxGxxG conserved consensus sequences within the Rossmann fold are underlined.

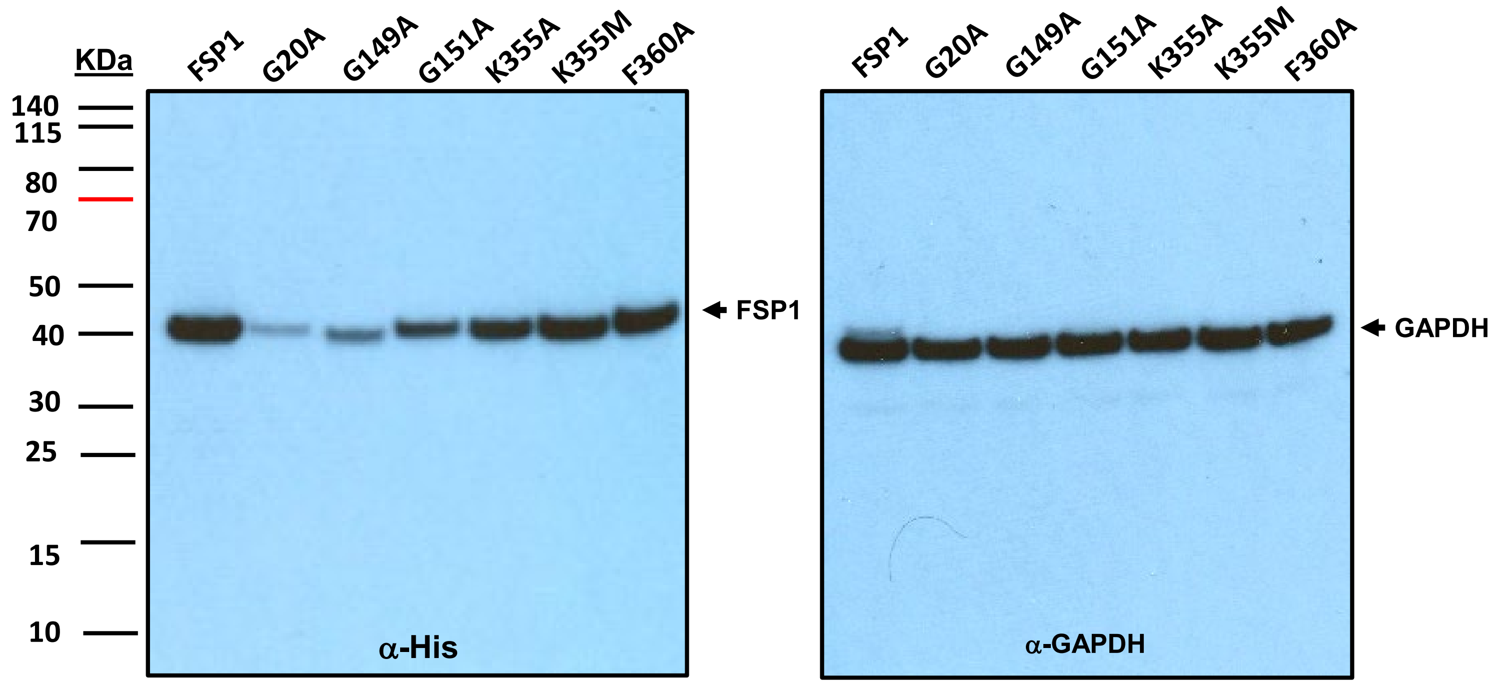

Supplementary Fig. 8 | Immunoblotting of FSP1 and its mutants with a His-tag at the C-terminus. His-Tagged FSP1 and its mutants were transiently expressed in HEK293 cells. Forty-eight hours post-transfection, transfected cells were harvested and the cell lysates were used for SDS-PAGE electrophoresis. Protein bands were transferred to PVDF membrane and probed by anti-His antibody (left) or anti-GADPH antibody as described in the Methods. Similar results were observed at least twice times as shown in these blots.

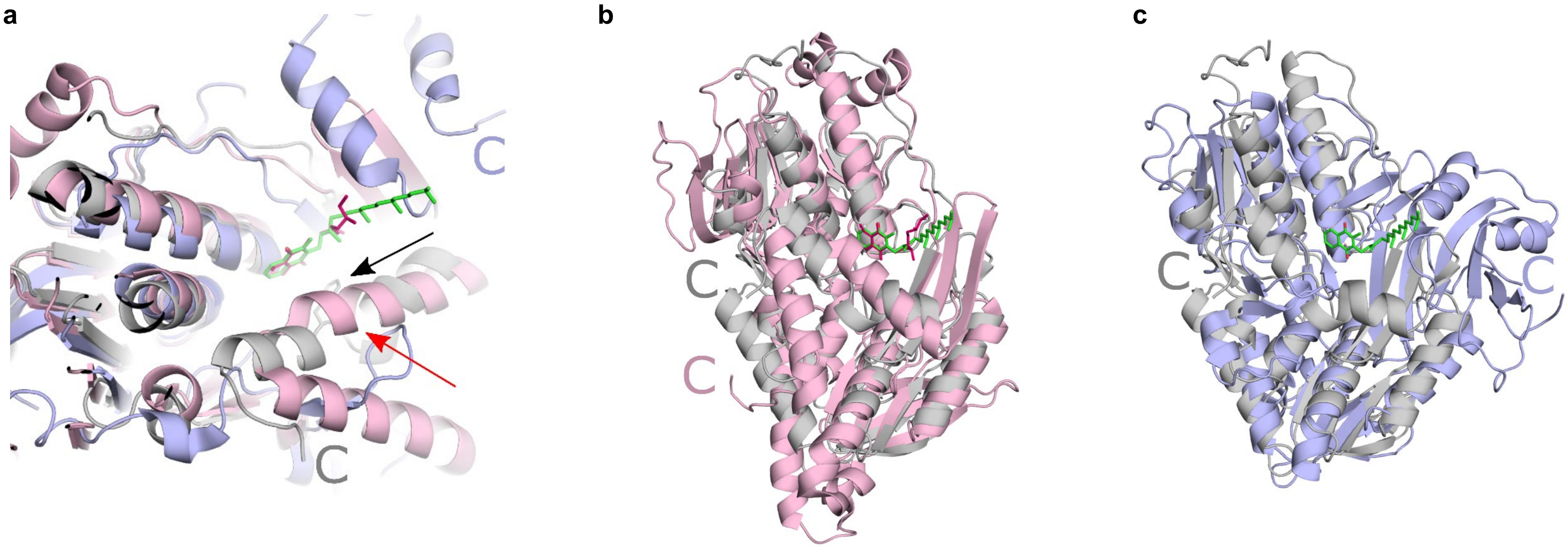

Supplementary Fig. 9 | Structure comparison of FSP1 and yeast Ndi1 and AIF. (a) Superpositions of Ndi1 (pink, PDBcode 4G73) with a bound quinone (raspberry) and apoptosis inducing factor (AIF, PDBcode 4BUR, cornflower blue) onto the AlphaFold model of FSP1 (grey) display differences in the C-termini's surrounding the putative vitamin K (green) binding site. Red arrow denotes deviations between the kinked C-terminal helix of the FSP1 model and the equivalent penultimate helix of Ndi1. The C-terminus of AIF is located away from this position. Black arrow denotes the position of residue F360 located near the vitamin K quinone moiety in the FSP1 model. (b) Global superposition of Nid1 structure with Alphafold model of FSP1. (c) Global superposition of 4BUR with Alphafold model of FSP1.

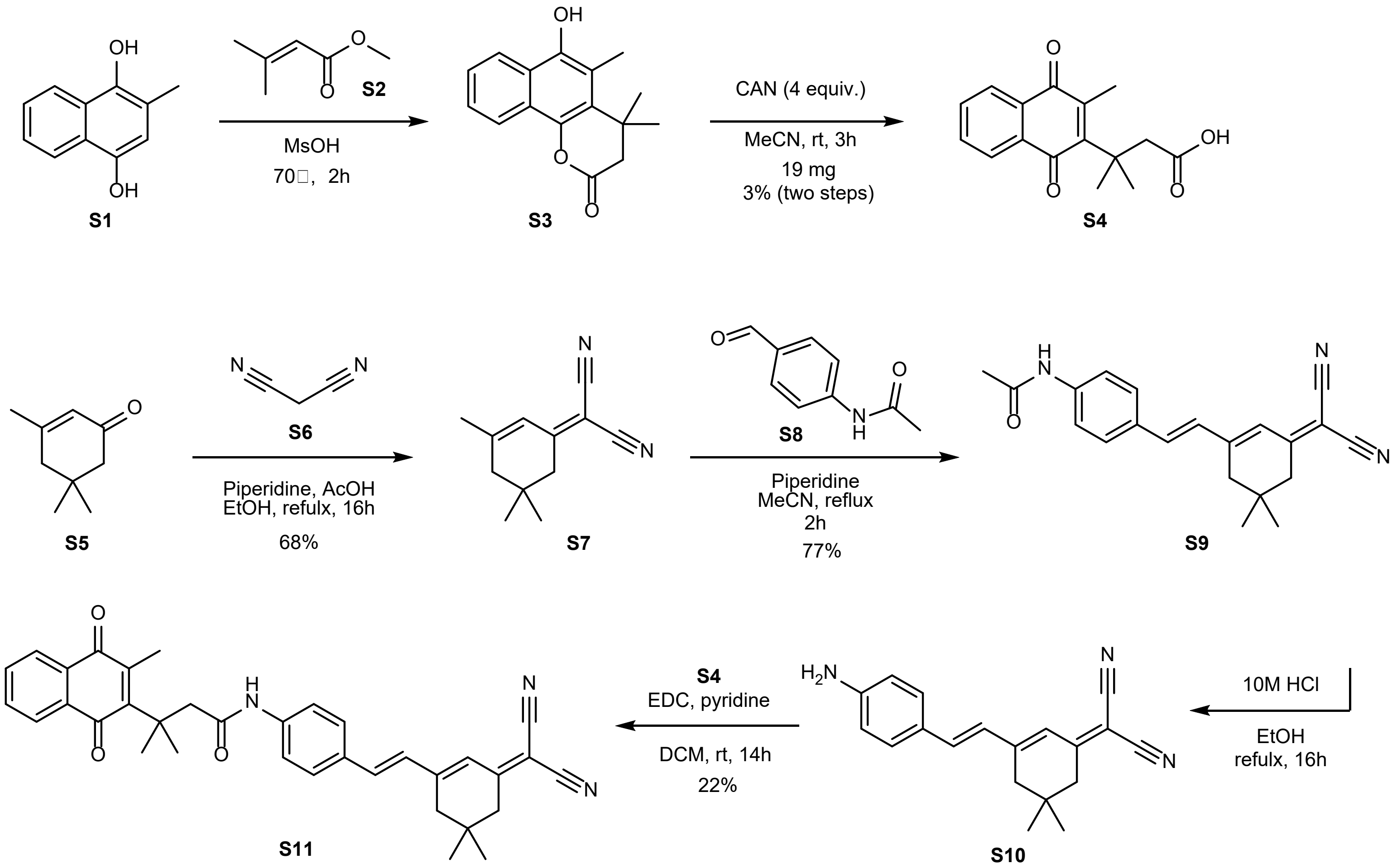

Supplementary Fig. 10 |Synthetic route for VK-ASM (S11).

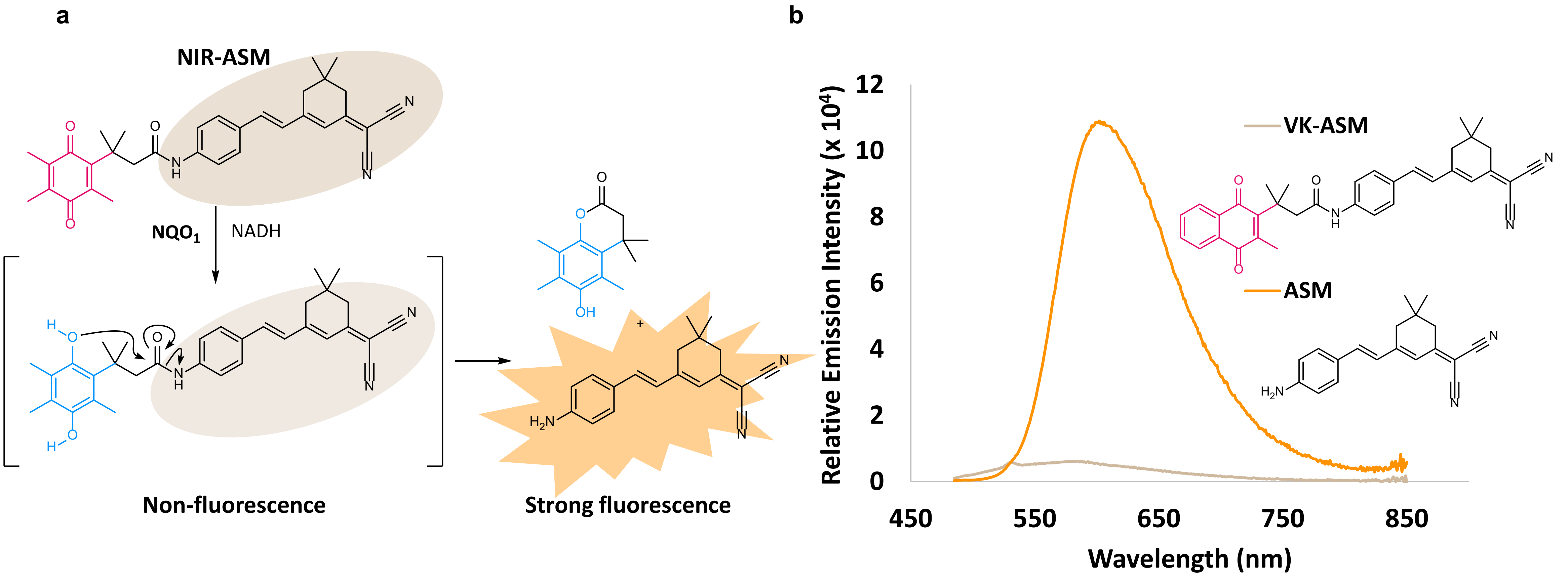

Supplementary Fig. 11 | Activity-based fluorescence probe of vitamin K and ubiquinone for FSP1. (**a**) Proposed activation mechanism of the activity-based fluorescent probe of ubiquinone (NIR-ASM) towards NQO1. The probe is inactive until the ubiquinone moiety is reduced to hydroquinone, and the rearrangement of the reduced intermediated releases the strong fluorescent tag ASM. (**b**) Fluorescence emission spectra of the fluorescence tag ASM (blue) and the activity-based probe of vitamin K before activation (orange) excited at 460 nm. Solution measurements were performed in 10×10 mm^2 quartz cuvettes. Photoluminescence spectra (PL) were measured using Edinburgh Instruments FS5 fluorescence spectrometer in steady-state mode with xenon lamp as the excitation source, exciting at 460 nm.

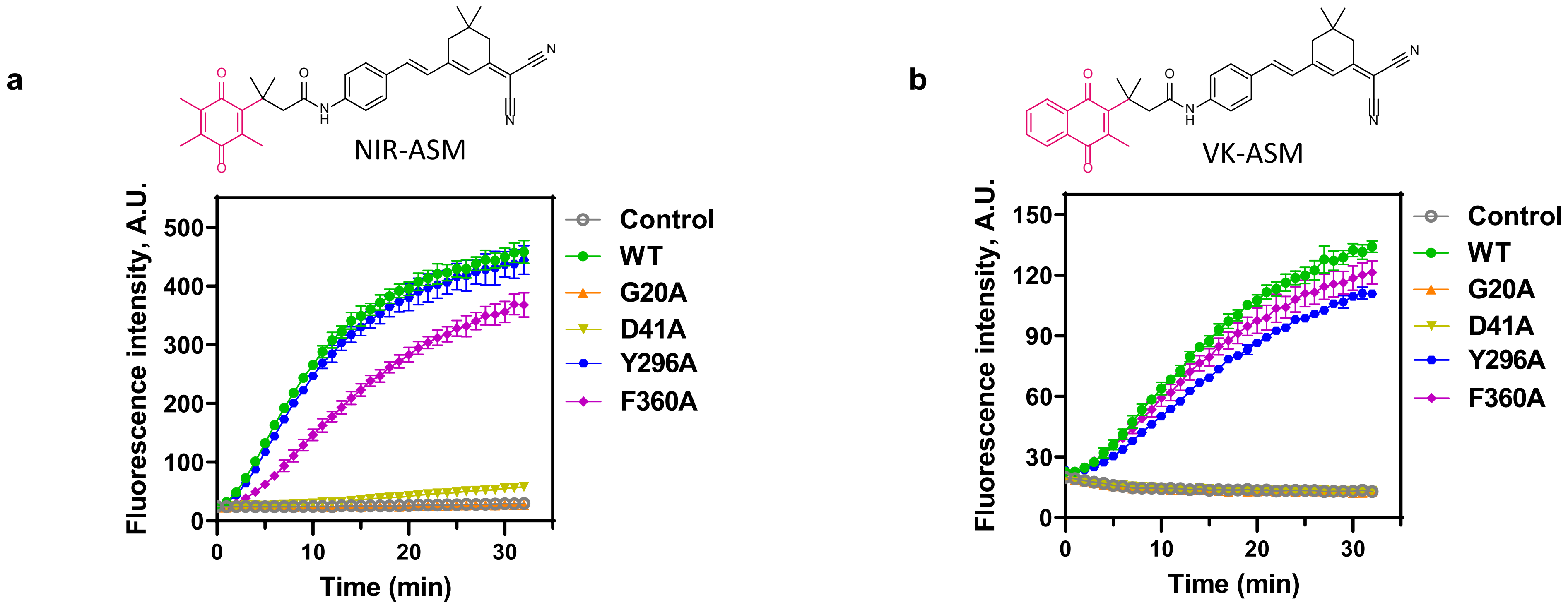

Supplementary Fig. 12 | Comparison of the effect of FSP1 mutations on activity-based fluorescence probes of NIR-ASM and VK-ASM. Wild-type FSP1 or its mutants were transiently expressed in TKO cells and the transfected cells were harvested 48 hours after transfection. Cell lysate was used for activity assays using the fluorescence probe NIR-ASM (**a**) and VK-ASM (**b**) for ubiquinone and vitamin K reduction, respectively, as described in the method section. Control: TKO cells without transfection. Data are presented as mean ± SD of three independent experiments (n=3).

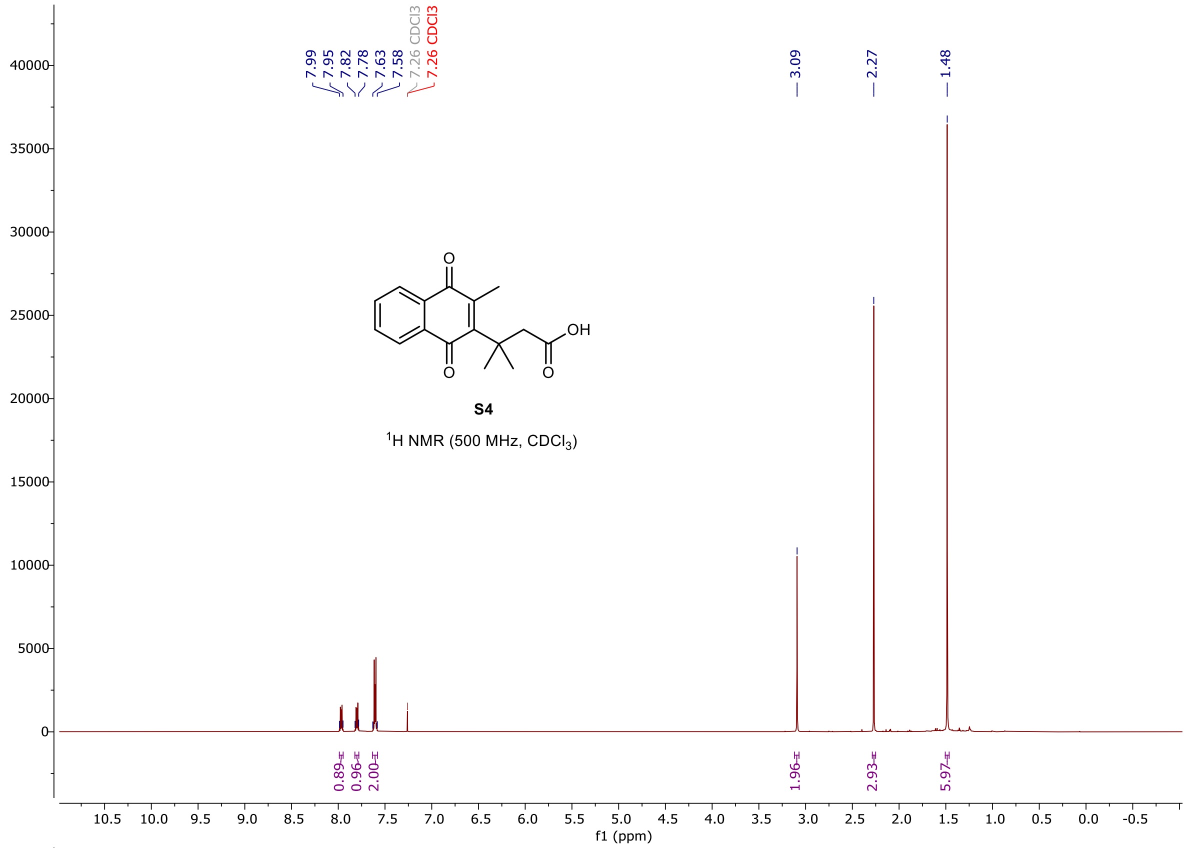

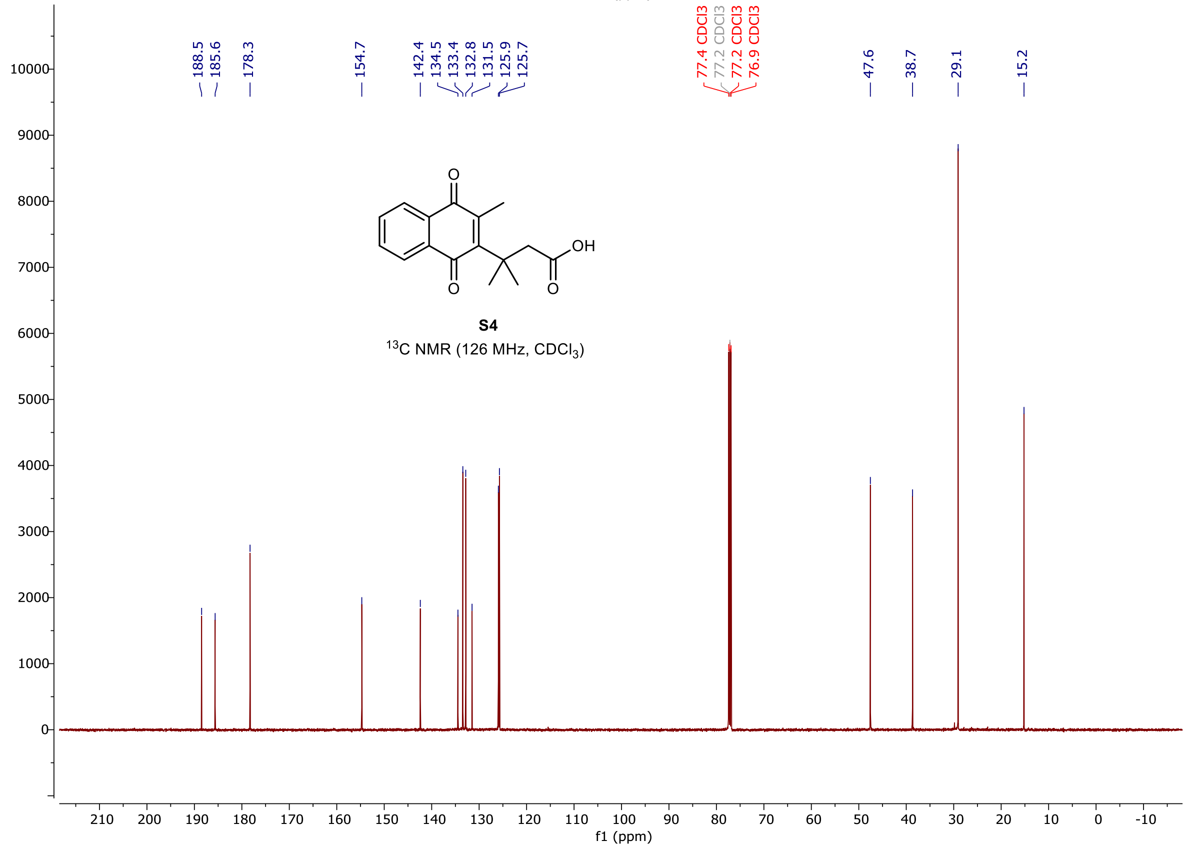

Supplementary Fig. 13 ^{1}H and ^{13}C NMR spectra of synthetic intermediates (S4, S7, S9) and VK-ASM (S11)

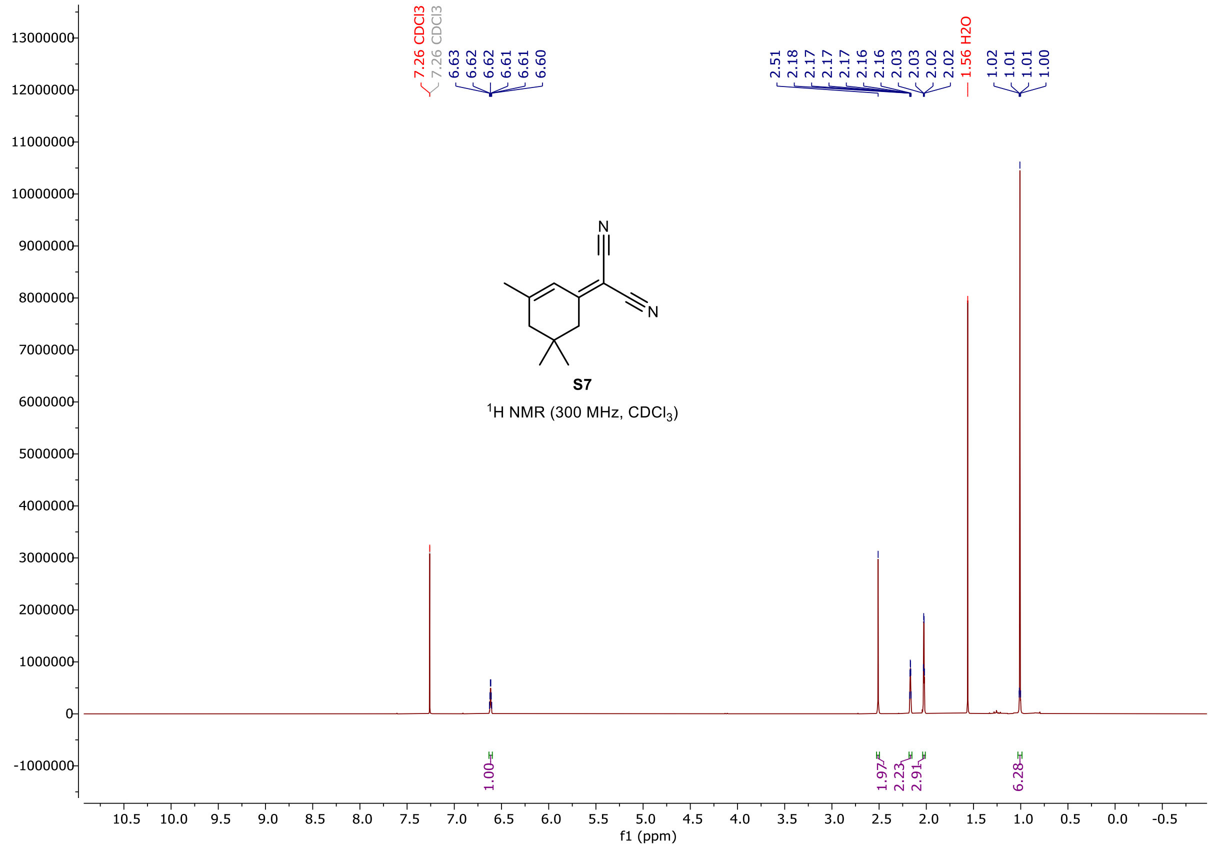

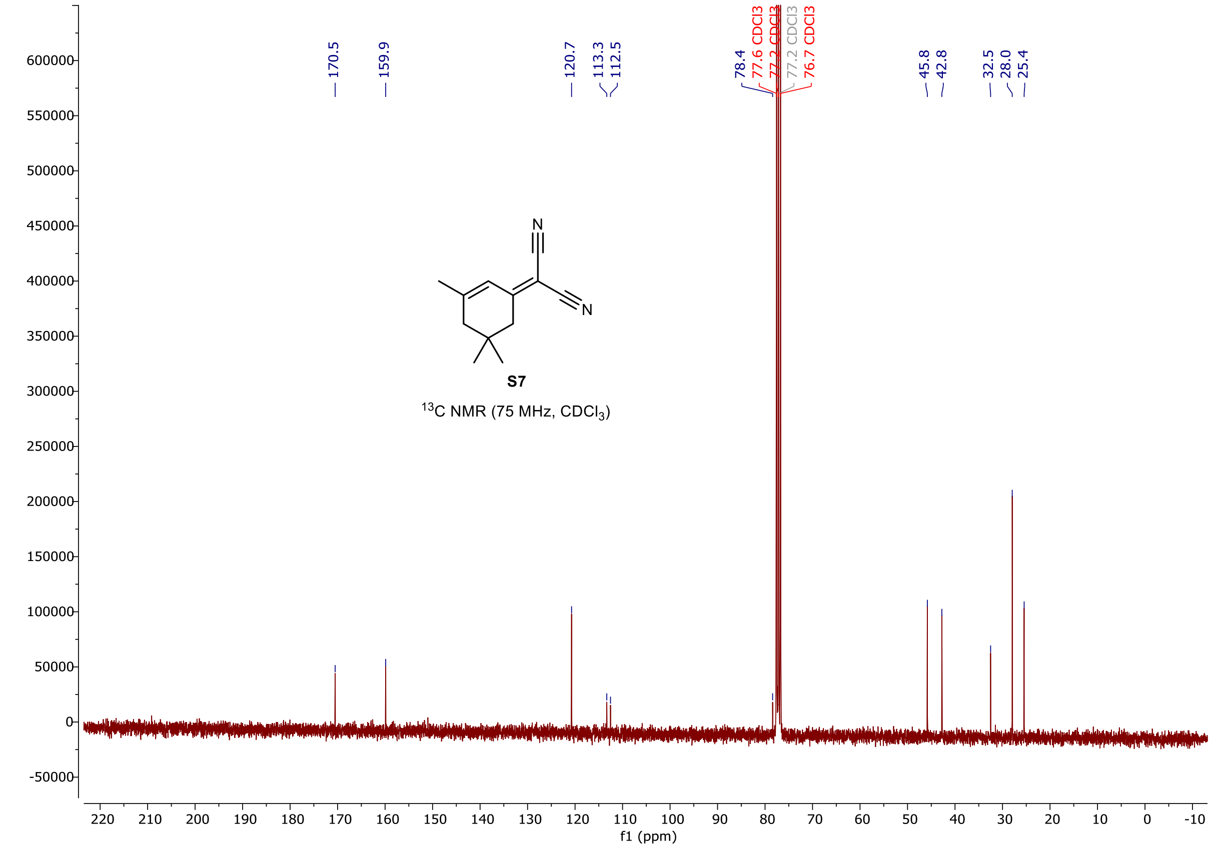

Supplementary Fig. 13 (continued)

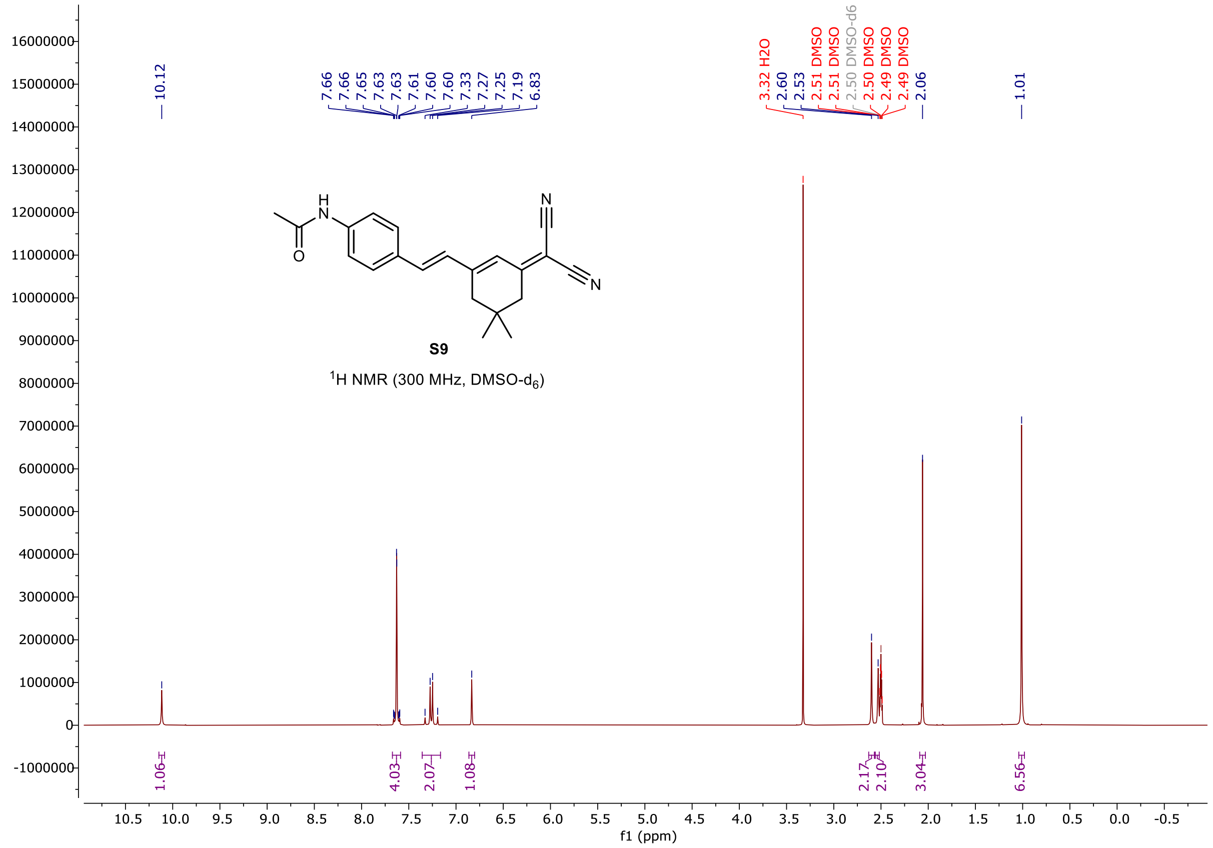

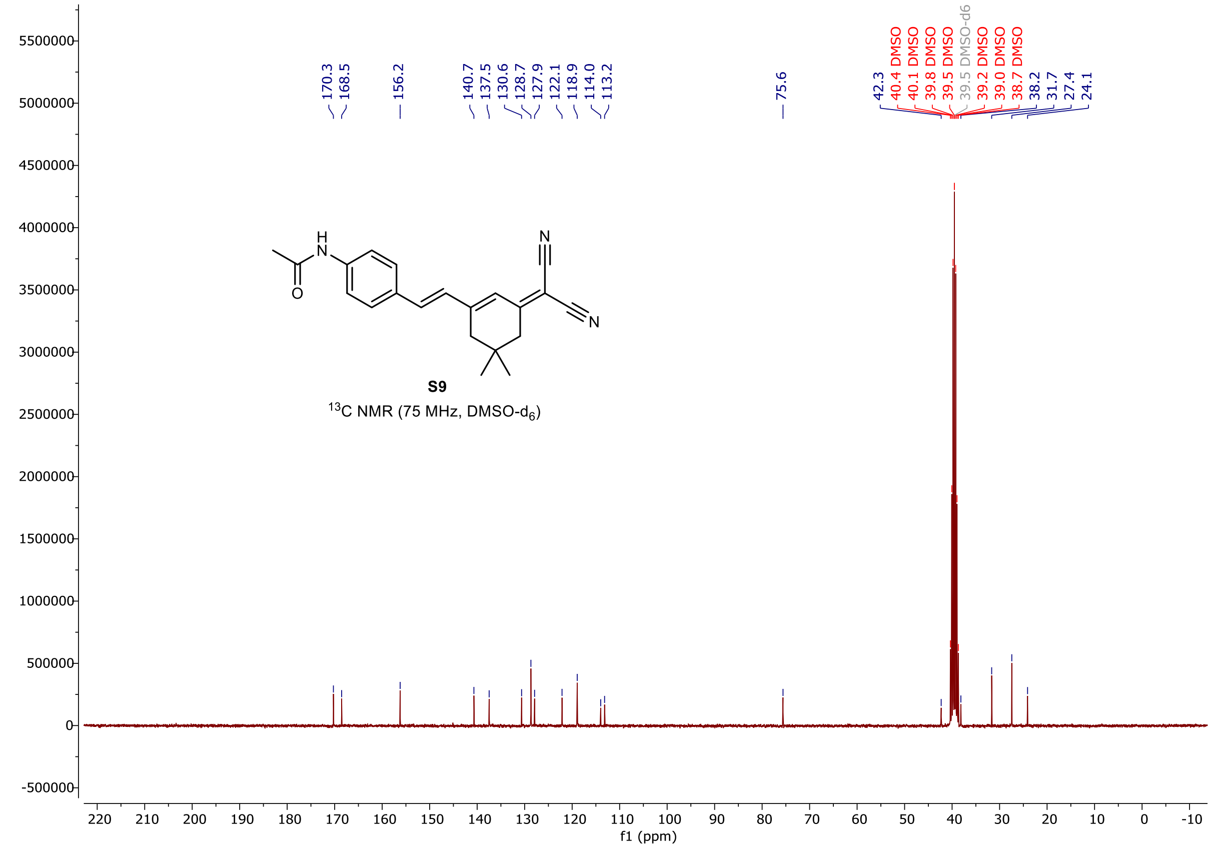

Supplementary Fig. 13 (continued)

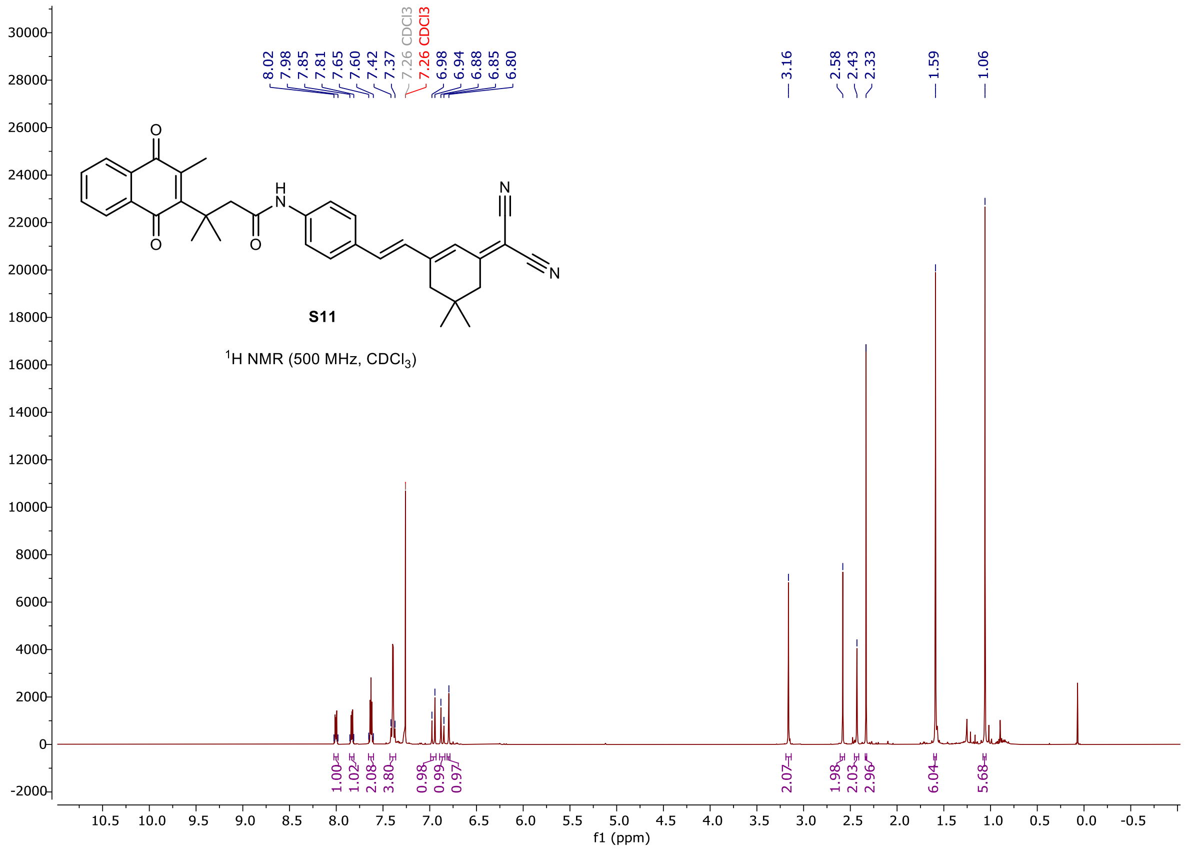

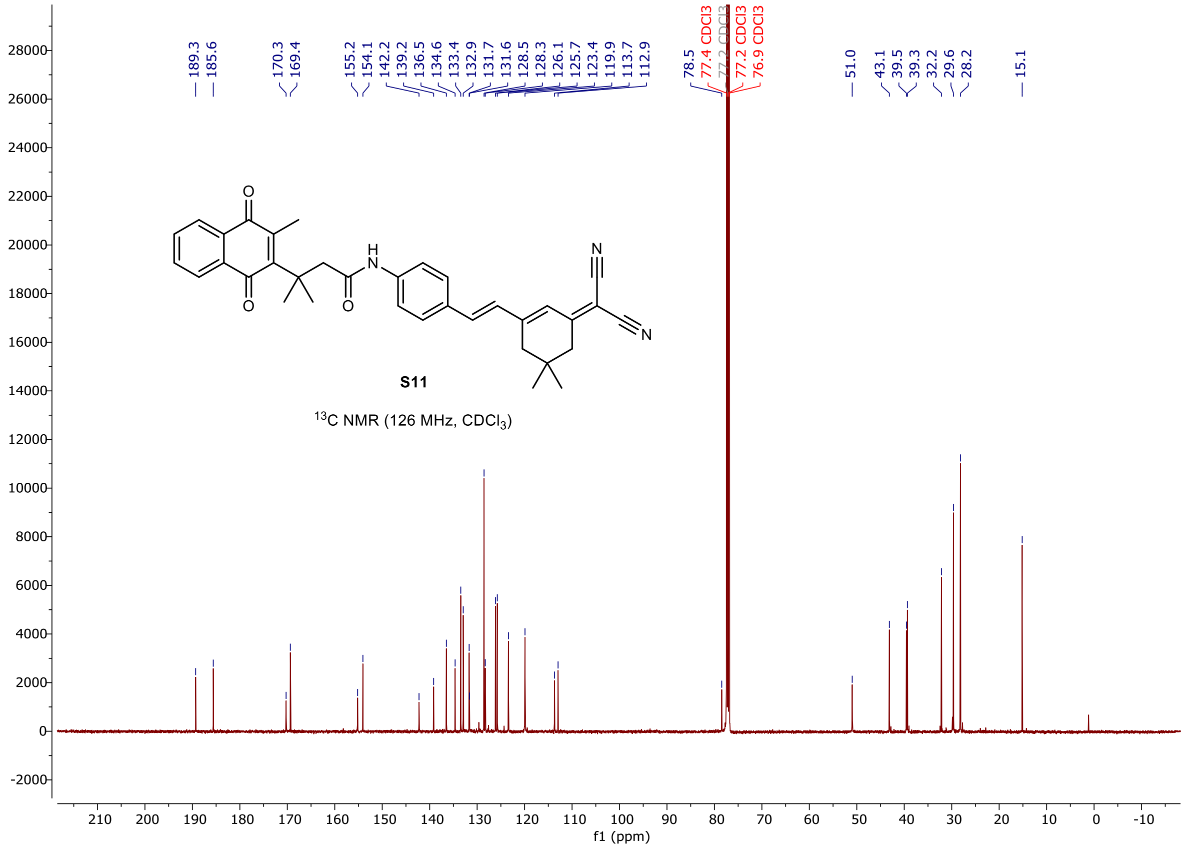

Supplementary Fig. 13 (continued)

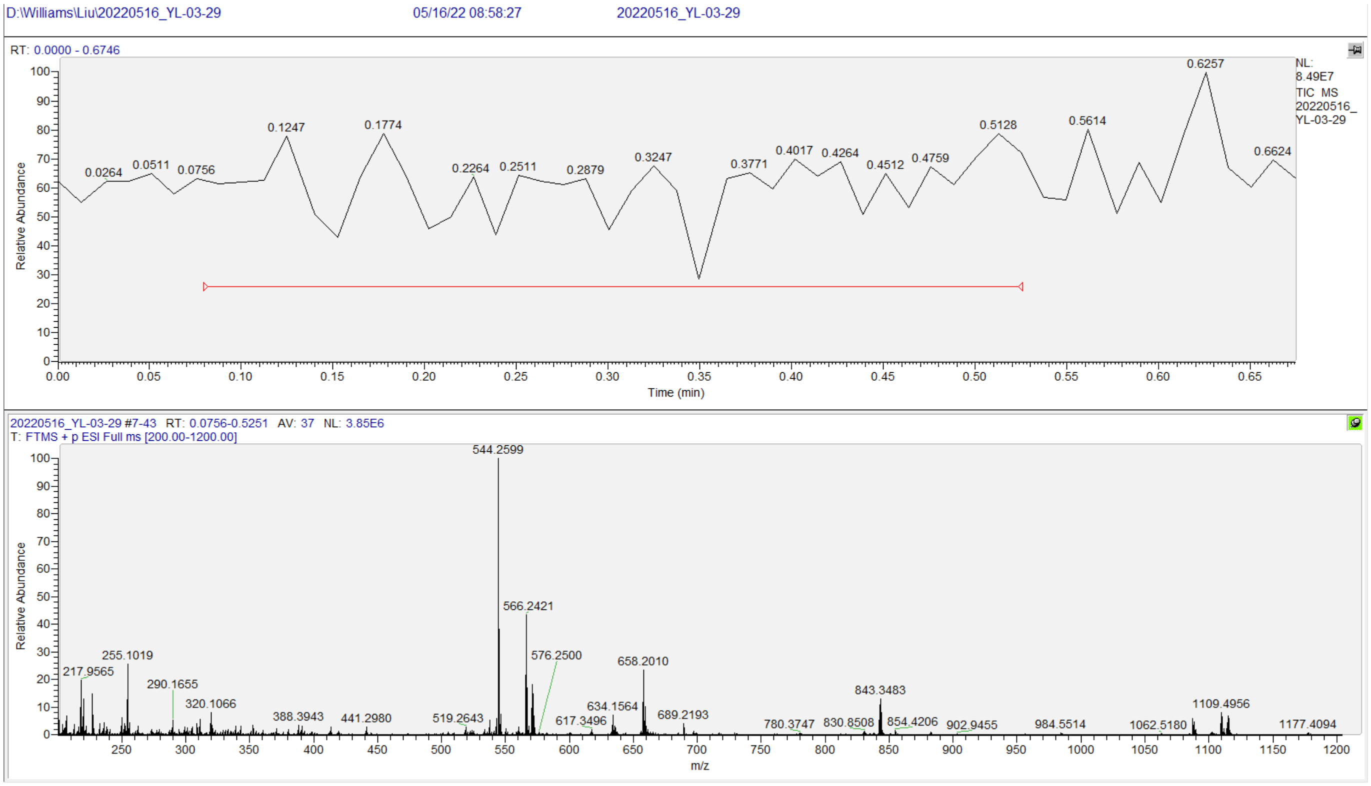

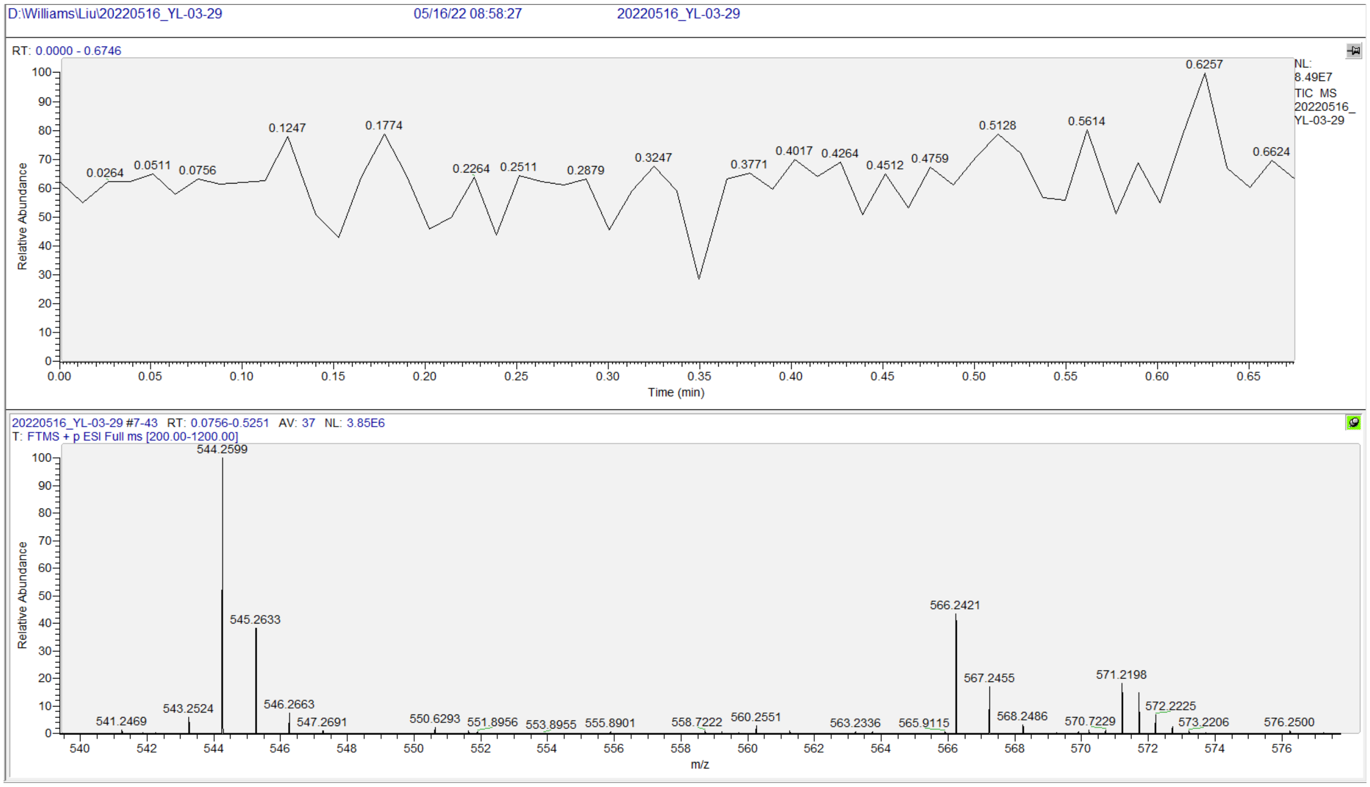

Supplementary Fig. 14 ESI MS Spectra of VK-ASM (S11)
