# Supplementary material for: A genome-wide CRISPR-Cas9 knockout screen identifies FSP1 as the warfarin-resistant vitamin K reductase
Source: Nat Commun. 2023 Feb 14;14:828. doi: 10.1038/s41467-023-36446-8 (PMC9929328; doi:10.1038/s41467-023-36446-8)
Supplement: Supplementary file 3 — Description of Additional Supplementary Files [file 41467_2023_36446_MOESM3_ESM.pdf]

# Description of Additional Supplementary Files

**Supplementary Data 1 |** Enrichment of sgRNA targeting genes related to cell proliferation, apoptosis proteins, and calcium-dependent proteins

**Supplementary Data 2 |** Top-hit potential candidates of warfarin-resistant VKR
