# Supplementary material for: A genome-wide CRISPR-Cas9 knockout screen identifies FSP1 as the warfarin-resistant vitamin K reductase
Source: Nat Commun. 2023 Feb 14;14:828. doi: 10.1038/s41467-023-36446-8 (PMC9929328; doi:10.1038/s41467-023-36446-8)
Supplement: Supplementary file 6 — Reporting Summary [file 41467_2023_36446_MOESM6_ESM.pdf]

nature portfolio

Corresponding author(s): Jian-Ke Tie

Last updated by author(s): Jan 11, 2023

Reporting Summary

Nature Portfolio wishes to improve the reproducibility of the work that we publish. This form provides structure for consistency and transparency in reporting. For further information on Nature Portfolio policies, see our Editorial Policies and the Editorial Policy Checklist.

Statistics

For all statistical analyses, confirm that the following items are present in the figure legend, table legend, main text, or Methods section.

n/a	Confirmed	
☐	☒	The exact sample size (*n*) for each experimental group/condition, given as a discrete number and unit of measurement
☐	☒	A statement on whether measurements were taken from distinct samples or whether the same sample was measured repeatedly
☒	☐	The statistical test(s) used AND whether they are one- or two-sided<br>*Only common tests should be described solely by name; describe more complex techniques in the Methods section.*
☒	☐	A description of all covariates tested
☐	☒	A description of any assumptions or corrections, such as tests of normality and adjustment for multiple comparisons
☐	☒	A full description of the statistical parameters including central tendency (e.g. means) or other basic estimates (e.g. regression coefficient) AND variation (e.g. standard deviation) or associated estimates of uncertainty (e.g. confidence intervals)
☒	☐	For null hypothesis testing, the test statistic (e.g. *F*, *t*, *r*) with confidence intervals, effect sizes, degrees of freedom and *P* value noted<br>*Give P values as exact values whenever suitable.*
☒	☐	For Bayesian analysis, information on the choice of priors and Markov chain Monte Carlo settings
☒	☐	For hierarchical and complex designs, identification of the appropriate level for tests and full reporting of outcomes
☒	☐	Estimates of effect sizes (e.g. Cohen's *d*, Pearson's *r*), indicating how they were calculated

Our web collection on statistics for biologists contains articles on many of the points above.

Software and code

Policy information about availability of computer code

Data collection	Next generation sequencing data collection was performed by Broad Institute Genomic Services. No other software was used in this study for data collection.
Data analysis	Coot (version 0.9.6) is a molecular graphics software used to model structures. In this case, to position the cofactors and substrate into the AlphaFold model.<br>PyMol (version 2.2.0) was used to generate the cartoon diagrams of the AlphaFold model with cofactors and substrate present.<br>GraphPad Prism v9 (GraphPad Software) was used for data analysis of most activity assays.<br>Cutadapt v. 2.9 was used for trimming the original sequence from NGS results. Bowtie2 v.2.4.1 was used for sequence alignment, Samtools v.1.13 was used for generating the counts of sgRNA using "idxstats" option.

For manuscripts utilizing custom algorithms or software that are central to the research but not yet described in published literature, software must be made available to editors and reviewers. We strongly encourage code deposition in a community repository (e.g. GitHub). See the Nature Portfolio guidelines for submitting code & software for further information.

Data

Policy information about availability of data

All manuscripts must include a data availability statement. This statement should provide the following information, where applicable:

- Accession codes, unique identifiers, or web links for publicly available datasets
- A description of any restrictions on data availability
- For clinical datasets or third party data, please ensure that the statement adheres to our policy

Alphafold model of FSP1 can be found at https://alphafold.ebi.ac.uk/entry/Q9BRQ8.
Coordinates for Ndi1 can be found at https://www.rcsb.org/structure/4G73.
Coordinates of the superposition of the substrates from coordinates 4G73 into the Alphafold model are available in the supplementary material.
Tissue specificity of FSP1 gene is available from The Human Protein Atlas Database at https://www.proteinatlas.org/ENSG00000042286-AIFM2/tissue.
The raw data and uncropped gel images are available in the Source Data file.

Human research participants

Policy information about studies involving human research participants and Sex and Gender in Research.

Reporting on sex and gender	N/A
Population characteristics	N/A
Recruitment	N/A
Ethics oversight	N/A

Note that full information on the approval of the study protocol must also be provided in the manuscript.

Field-specific reporting

Please select the one below that is the best fit for your research. If you are not sure, read the appropriate sections before making your selection.

☒ Life sciences ☐ Behavioural & social sciences ☐ Ecological, evolutionary & environmental sciences

For a reference copy of the document with all sections, see nature.com/documents/nr-reporting-summary-flat.pdf

Life sciences study design

All studies must disclose on these points even when the disclosure is negative.

Sample size	For genome-wide CRISPR-Cas9 screening, sample size was determined based on the library size of sgRNAs to ensure a coverage of >500 cells expressing each sgRNA according to reference 17. For all other experiments, no statistical estimation of sample size was performed, and the sample sizes are chosen because they provide sufficient confidence to assess the experimental results.
Data exclusions	No data were excluded from the analysis.
Replication	Considering the costly next generation sequencing experiment, two independent genome-wide knockout screening were performed. For all cell-based functional studies, triplicates were performed to obtain a confident results and were presented as mean with standard derivation. For western blot analysis, at least two replicates were performed and yielded consistent results.
Randomization	No randomized experiment was performed in this study.
Blinding	Blinding was not relevant for this study since no group allocation was performed

Reporting for specific materials, systems and methods

We require information from authors about some types of materials, experimental systems and methods used in many studies. Here, indicate whether each material, system or method listed is relevant to your study. If you are not sure if a list item applies to your research, read the appropriate section before selecting a response.

Materials & experimental systems

n/a	Involved in the study
☐	☒ Antibodies
☐	☒ Eukaryotic cell lines
☒	☐ Palaeontology and archaeology
☒	☐ Animals and other organisms
☒	☐ Clinical data
☒	☐ Dual use research of concern

Methods

n/a	Involved in the study
☒	☐ ChIP-seq
☒	☐ Flow cytometry
☒	☐ MRI-based neuroimaging

Antibodies

Antibodies used	Mouse anti-FIXgla (Green Mountain Antibodies, Clone BC2, #GMA 001)<br>Rabbit anti-AIFM2/FSP1 (Cell Signaling Technology, #24972)<br>Rabbit anti-AIFM2 (Sigma, #ZRB1527, Clone 1I15, lot Q3709968)<br>Mouse anti-Fas (Cell Signaling Technology, #8023, clone 4C3)<br>Mouse anti-Caspase-8 (Cell Signaling Technology, #9746, clone 1C12)<br>Sheep anti-human Protein C (Affinity Biologicals, SAPC-AP, lot AP3079-ER2)<br>HRP-conjugated sheep anti-human Protein C (Affinity Biologicals, SAPC-HRP, lot HRP1608-2R1)<br>HRP-conjugated mouse anti-His (ProteinTech, HRP-66005, Clone 1B7G5, lot 21000118)<br>Mouse anti-GAPDH (ProteinTech, 60004-1-Ig, Clone 1E6D9, lot 10004129).<br>Mouse anti-HPC4 (2mg/ml, home-made antibody was a gift from Dr. Esmon's lab at Oklahoma Medical Research Foundation)
Validation	Mouse anti-FIXgla antibody was validated by Green Mountain Antibodies for ELISA and Western blot. Information available at https://greenmoab.com/product/gma-001/.<br><br>Rabbit anti-AIFM2/FSP1 antibody was validated by Cell Signaling Technology for Western blot. Information available at https://www.cellsignal.com/products/primary-antibodies/aifm2-fsp1-antibody/24972.<br>Rabbit anti-AIFM2 antibody was validated by Sigma for immunocytochemistry, Western blot, affinity binding assay, and flow cytometry. Full information available at https://www.sigmaaldrich.com/US/en/product/sigma/zrb1527.<br><br>Mouse anti-Fas antibody was validated by Cell Signaling Technology for Western blot, immunofluorescent analysis, and flow cytometric analysis. Full information available at https://www.cellsignal.com/products/primary-antibodies/fas-4c3-mouse-mab/8023.<br>Mouse anti-Caspase-8 antibody was validated by Cell Signaling Technology for Western blot. Full information available at https://www.cellsignal.com/products/primary-antibodies/caspase-8-1c12-mouse-mab/9746.<br><br>Sheep anti-human Protein C polyclonal antibody and its HRP conjugate was validated by Affinity Biologicals for Species Cross Reactivity. Full information available at https://affinitybiologicals.com/species-cross-reactivity/.<br>HRP-conjugated mouse anti-His antibody was validated by ProteinTech for Western blot. Information available at https://www.ptglab.com/products/6-His,-His-Tag-Antibody-HRP-66005.htm.<br><br>Mouse anti-GAPDH antibody was validated by ProteinTech for Western blot, immunoprecipitation, immunofluorescent analysis, and flow cytometry. Full information available at https://www.ptglab.com/products/GAPDH-Antibody-60004-1-Ig.htm.<br>Mouse anti-HPC4 monoclonal antibody recognizes a 12 amino acid sequence (EDQVDPRLIDGK) is now commercially available from GenScript that has been validated for Western blot, immunoprecipitation, flow cytometry, immunocytochemistry/immunofluorescence, and ELISA. Full information available at https://www.genscript.com/antibody/A01774-THE_Protein_C_Tag_Antibody_HPC4_mAb_Mouse.html.

Eukaryotic cell lines

Policy information about cell lines and Sex and Gender in Research

Cell line source(s)	The human embryonic kidney 293 cell (HEK293) was obtained from ATCC (#CRL-1573).
Authentication	The HEK293 cell line used in this study has been verified by the Tissue Culture Facility of the University of North Carolina at Chapel Hill.
Mycoplasma contamination	All cell lines used in this study were tested negative for mycoplasma contamination.
Commonly misidentified lines<br>(See ICLAC register)	No cross-contamination was reported for HEK293 cell line used in this study according to the Tissue Culture Facility of the University of North Carolina at Chapel Hill.
